# Environmental variability and population dynamics: do European and North American ducks play by the same rules?

**DOI:** 10.1002/ece3.2413

**Published:** 2016-09-09

**Authors:** Hannu Pöysä, Jukka Rintala, Douglas H. Johnson, Jukka Kauppinen, Esa Lammi, Thomas D. Nudds, Veli‐Matti Väänänen

**Affiliations:** ^1^ Natural Resources Institute Finland Joensuu Finland; ^2^ Natural Resources Institute Finland Helsinki Finland; ^3^ USGS Northern Prairie Wildlife Research Center St. Paul MN USA; ^4^ Fisheries, Wildlife, and Conservation Biology University of Minnesota St. Paul MN USA; ^5^ Kuopio Natural History Museum Kuopio Finland; ^6^ Environmental Planning ENVIRO Espoo Finland; ^7^ Department of Integrative Biology University of Guelph Guelph ON Canada; ^8^ Department of Forest Sciences University of Helsinki Helsinki Finland

**Keywords:** demographic stochasticity, density dependence, environmental variability, hierarchical Bayesian state‐space models, life history strategy, population variability

## Abstract

Density dependence, population regulation, and variability in population size are fundamental population processes, the manifestation and interrelationships of which are affected by environmental variability. However, there are surprisingly few empirical studies that distinguish the effect of environmental variability from the effects of population processes. We took advantage of a unique system, in which populations of the same duck species or close ecological counterparts live in highly variable (north American prairies) and in stable (north European lakes) environments, to distinguish the relative contributions of environmental variability (measured as between‐year fluctuations in wetland numbers) and intraspecific interactions (density dependence) in driving population dynamics. We tested whether populations living in stable environments (in northern Europe) were more strongly governed by density dependence than populations living in variable environments (in North America). We also addressed whether relative population dynamical responses to environmental variability versus density corresponded to differences in life history strategies between dabbling (relatively “fast species” and governed by environmental variability) and diving (relatively “slow species” and governed by density) ducks. As expected, the variance component of population fluctuations caused by changes in breeding environments was greater in North America than in Europe. Contrary to expectations, however, populations in more stable environments were not less variable nor clearly more strongly density dependent than populations in highly variable environments. Also, contrary to expectations, populations of diving ducks were neither more stable nor stronger density dependent than populations of dabbling ducks, and the effect of environmental variability on population dynamics was greater in diving than in dabbling ducks. In general, irrespective of continent and species life history, environmental variability contributed more to variation in species abundances than did density. Our findings underscore the need for more studies on populations of the same species in different environments to verify the generality of current explanations about population dynamics and its association with species life history.

## 1 Introduction

Environmental variation and density dependence are key phenomena governing population dynamics (Hixon, Pacala, & Sandin, 2002; Murdoch, 1994; Royama, 1992; Sinclair & Pech, 1996; Turchin, 1995), and the role of the latter in particular has been studied extensively with ecological time series data (e.g., Brook & Bradshaw, 2006; Knape & de Valpine, 2012; Sibly, Barker, Denham, Hone, & Pagel, 2005; Turchin & Taylor, 1992; Woiwod & Hanski, 1992; Zeng, Nowierski, Taper, Dennis, & Kemp, 1998; Ziebarth, Abbott, & Ives, 2010). Relationships between them and variation in population size have received less attention. It has been suggested that low temporal variability in population size indicates density‐dependent regulation (e.g., Gaston & McArdle, 1994; Hanski, 1990). However, the few empirical studies that have addressed this relationship provided mixed results (see Hanski, 1990; Hanski & Woiwod, 1993; Holyoak & Baillie, 1996a; Williams, Ives, & Applegate, 2003). Irrespective of the possible relationship between density‐dependent regulation and temporal variation in population size, both theory (e.g., Kaitala, Ylikarjula, Ranta, & Lundberg, 1997; Ranta, Lundberg, Kaitala, & Laakso, 2000; Roughgarden, 1975) and laboratory experiments (e.g., Benton, Lapsley, & Beckerman, 2002; Laakso, Löytynoja, & Kaitala, 2003; Petchey, 2000) suggest that environmental variability affects temporal variation in population size. Theoretical work indicates that the outcome of this effect depends on the temporal autocorrelation of the environment and the responsiveness of the species to environmental fluctuations. In general, an undercompensating population (slow response to environmental change) suffers in slowly changing environments if a run of several bad years occurs, whereas an overcompensating population (rapid response to environmental change) suffers in environments that change dramatically from one time step to the next (see Ripa & Heino, 1999; Roughgarden, 1975; Schwager, Johst, & Jeltsch, 2006).

Generally, temporal environmental variability, or environmental stochasticity, has been considered an important factor affecting population dynamics, sometimes masking any signal of density‐dependent regulation (examples in Bonenfant et al., 2009; but see Herrando‐Pérez, Delean, Brook, Cassey, & Bradshaw, 2014). Gaston and McArdle (1994) reasoned that, in variable environments, populations may exhibit high temporal variability regardless of the operation of density‐dependent regulation. Even though environmental stochasticity might drive population dynamics, populations are not necessarily in a nonequilibrium or unregulated state (Sinclair & Pech, 1996; for a concise review of the debate about population regulation and environmental variability, see Turchin, 1995). It has been generally acknowledged that both density dependence and environmental stochasticity are important and often interact in affecting population dynamics (e.g., Bjørnstad & Grenleff, 2001; Ross, Hooten, DeVink, & Koons, 2015; Turchin, 1995, 1999), although their relative roles are less often clear. Depending on the relative strength of density dependence versus environmental stochasticity, there will be a continuum of dynamics ranging from tight population regulation around a stable equilibrium to totally stochastic dynamics (Turchin, 1995).

Considering the importance of environmental stochasticity and different population processes in affecting the ability of a species to respond to changes in environmental variability, information about the relationships between them from natural systems is surprisingly scant (see also Benton et al., 2002). One of the few examples of the influence of environmental variability on population dynamics comes from North American prairies, where the number of wetlands, a critical resource for breeding ducks, varies drastically between years (e.g., Batt, Anderson, Anderson, & Caswell, 1989). Such variation affects the local settlement and numbers of breeding pairs (Johnson & Grier, 1988), causes drought‐induced emigration of breeding ducks from prairies to less variable mixed‐prairie and parkland regions (Johnson & Grier, 1988; see also Bethke, 1993; Bethke & Nudds, 1995) and drives spatial synchrony in breeding numbers of ducks (Drever, 2006). Several earlier studies suggested that environmental variability plays a central role in affecting the variability of duck populations and the strength of density‐dependent population regulation. For example, Nudds (1983) demonstrated that duck populations are more variable in mixed‐prairie habitats than in relatively more stable aspen parkland habitats of the Canadian prairies. In addition, Vickery and Nudds (1984) found that dabbling ducks, which occupy the most temporally variable wetlands in the mixed‐prairie habitats, showed less evidence of density‐dependent regulation than did diving ducks, which use temporally more stable wetlands. This finding was accompanied by later work analyzing density dependence in time series of North American ducks (Jamieson & Brooks, 2004). A general view is that dabbling ducks respond more readily to changes in wetland conditions than do diving ducks (but see Leitch & Kaminski, 1985) and, hence, exhibit less density dependence in population regulation, a distinction reflecting differences in life history between dabbling and diving ducks along a “slow–fast continuum” sensu Sæther (1987; based on the r/K scheme presented by Pianka, (1970)) (Bailey, 1981; Gunnarsson et al., 2013; Johnson & Grier, 1988; Nummi, Holopainen, Rintala, & Pöysä, 2015; Péron, Nicolai, & Koons, 2012; Vickery & Nudds, 1984; Viljugrein, Stenseth, Smith, & Steinbakk, 2005).

Murray, Anderson, and Steury (2010), however, did not find support for the idea that the strength of density dependence is associated with species life history in North American ducks. Furthermore, recent analyses have reached mixed conclusions about the prevalence of density‐dependent regulation in North American ducks altogether (see also Gunnarsson et al., 2013). Sæther et al. (2008) concluded that weak density regulation is a general characteristic of the population dynamics of North American mid‐continental duck species. On the other hand, Murray et al. (2010) found evidence of density‐dependent regulation in all seven dabbling duck species and in three diving duck species studied, but a reanalysis of time‐segmented data revealed that density‐dependent regulation was weaker in 1980–2005 than it had been during 1955–1979 for both dabbling and diving ducks. Lawrence, Gramacy, Thomas, and Buckland (2013) in turn concluded that there is little evidence of density‐dependent regulation in many North American duck species. A similar conclusion was reached by Roy, McIntire, and Cumming (2016) for mallards (*Anas platyrhynchos* Linnaeus, a dabbling duck) breeding across western North America, ranging from the highly variable Prairie Pothole Region to the more stable western boreal forests. In addition, these authors found that different environmental factors affected population growth rates in different regions. Ross et al. (2015) reported that, irrespective of climate‐driven variability in environmental conditions, density dependence was an important driver of population dynamics in scaup (two species of diving ducks, *Aythya* spp.) in the northwest territory regions of Canada. Finally, Feldman, Anderson, Howerter, and Murray (2015) found that, while the effect of environmental stochasticity on duck population dynamics was weak at the core of the Prairie Pothole Region, it was strongly influential in peripheral sites, but species response varied by site.

**Figure 1:**
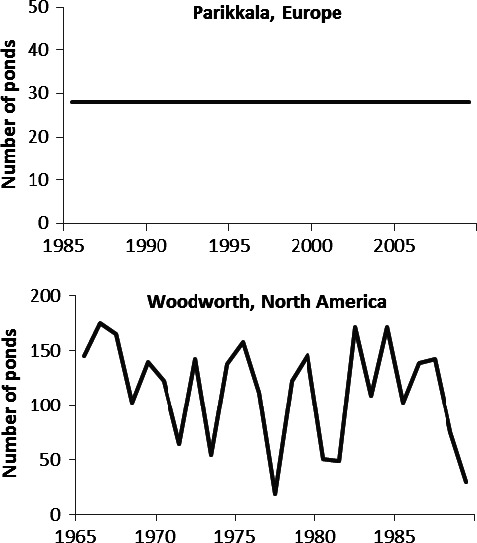
Examples showing the difference between northern Europe and North American prairies in the variability of duck breeding environments (the number of wetland basins/ponds containing water)

To clarify the role of environmental variability in population dynamics, we need more empirical studies from systems in which masking effects of spatial correlation are absent and where temporal shifts in environmental variability do not dominate. There is a fundamental difference between North American prairies and northern European boreal areas in the variability of breeding environments of ducks: The number of wetlands varies drastically between years on the prairies, but remains essentially stable in northern Europe (examples in Fig. 1). Further, habitat conditions in north European lakes (e.g., structure of shore vegetation and water level) show little short‐ or long‐term variation, except that caused by beaver dams (Holopainen, Nummi, & Pöysä, 2014; Nummi & Pöysä, 1993; Suhonen, Nummi, & Pöysä, 2011). This being the case, effects on duck numbers from such short‐term environmental variation, as described above for North American ducks, should be less important in northern Europe than in North American prairies. Hence, because the same duck species, or close ecological counterparts, occur in both North America and Europe (see Methods), a comparison between continents offers a unique opportunity to study whether and how the magnitude of density effects might vary in comparison with the effects of environmental variability on population dynamics.

We developed hierarchical Bayesian (Gelman, Carlin, Stern, & Rubin, 2003) state‐space models (Dennis, Ponciano, Lele, Taper, & Staples, 2006; Mutshinda, O'Hara, & Woiwod, 2011) of yearly dynamics of six species of dabbling ducks (*Anas* spp.) and two species of diving ducks (*Aythya* spp.) in each of Europe and North America. Hierarchical approaches are strongly recommended for estimating, for instance, the importance of density‐dependent regulation of populations (Lebreton & Gimenez, 2013). We tested two predictions derived from theoretical work on the importance of environmental variability in temporal population dynamics and from the earlier findings on North American ducks. First, considering the drastic difference in environmental variability between northern Europe and North America, we expected density dependence to govern duck population dynamics in northern Europe and environmental variability in North America. Second, if species’ life history strategies further mediate population dynamical responses to environmental variability, there should be less difference between dabbling (presumed “fast species”) and diving (presumed “slow species”) ducks in the relative importance of density dependence where environmental variability is lower, that is, in northern Europe versus North America. We also modeled the effect of demographic stochasticity on population dynamics because it is an important component that may affect particularly small populations (e.g., Lande, 1993; Lande, Engen, & Sæther, 2003). To our knowledge, no earlier work has compared population dynamics of a group of noncyclic species in such a setting, that is, the same species (or ecological counterparts) between continents in contrasting environments (for species with cyclic dynamics, see Stenseth, 1999). Hence, our study provides a novel approach to address the importance of environmental variability in population dynamics in general.

## 2 Methods

### 2.1 Population data

The data include 56 time series (23–33 years) of breeding numbers of eight duck species from four study areas in Finland (all have stable wetland conditions typical of northern Europe) and 16 time series of breeding numbers of eight duck species from the Redvers Waterfowl Study Area in Saskatchewan, Canada (eight time series of 26 years, data from Vickery & Nudds, 1984), and from the Woodworth Study Area in North Dakota, USA (eight time series of 25 years, data from Johnson, 1995), representing variable wetland conditions of North American prairies (Table S1). Throughout the text, one time series means the numbers of breeding pairs of one species at one site. We included eight species pairs (i.e., the same species or matched species [close ecological counterparts] from Europe and North America) for which we had time series from both continents: Eurasian Wigeon (*Anas penelope* Linnaeus; Europe) and American Wigeon (*Anas americana* Gmelin; North America); Mallard (Europe and North America); Northern Shoveler (*Anas clypeata* Linnaeus; Europe and North America); Northern Pintail (*Anas acuta* Linnaeus; Europe and North America); Garganey (*Anas querquedula* Linnaeus; Europe) and Blue‐winged Teal (*Anas discors* Linnaeus; North America); Eurasian Teal/Green‐winged Teal (*Anas crecca* Linnaeus; Europe and North America); Common Pochard (*Aythya ferina* Linnaeus; Europe) and Redhead (*A. americana* Eyton; North America); Tufted Duck (*Aythya fuligula* Linnaeus; Europe) and Lesser Scaup (*A. affinis* Eyton; North America). In most of the matched species cases, the species also are phylogenetically close relatives (Gonzalez, Dűttmann, & Wink, 2009); Garganey and Blue‐winged Teal are not the closest relatives but are ecologically very similar (e.g., Nudds, Sjöberg, & Lundberg, 1994). As to the published time series, we refer to the original articles for study areas and methodological details (Johnson, 1995; Vickery & Nudds, 1984).

All of the Finnish time series are based on ground surveys carried out by one of us in the respective study regions, using the standard methods for monitoring breeding numbers of ducks in Finland (census methods described in detail in Koskimies & Väisänen, 1991). In brief, to take into account differences in the timing of spring migration between species, 2–4 censuses were carried out in May in each study region; pair numbers for a species were interpreted using the field observations from the census within the recommended species‐specific time window (Kauppinen, 1983; Koskimies & Väisänen, 1991; Pöysä, 1996).

The Finnish time series are from four regions: (1) Hollola in southern Finland (61°N, 25°E; two isolated lakes 7 km apart and a group of two lakes 0.1 km apart; duck surveys carried out by E. Lammi); (2) Parikkala in southeast Finland (61°N, 29°E; a group of 28 lakes, all within 6 × 7 km; duck surveys carried out by H. Pöysä); (3) Pieksämäki‐Suonenjoki‐Kuopio‐Siilinjärvi in Central Finland (hereafter, Kuopio, 62°N, 27°E; six isolated lakes with mean distance to closest neighbor 15.6 km, range 11.3–45.1 km; duck surveys carried out by J. Kauppinen); and (4) Maaninka in Central Finland (63°N, 27°E; a group of four lakes within 5 × 6 km; distance to nearest neighbor 0.9–3.2 km; duck surveys carried out by V.‐M. Väänänen). Time series from isolated lakes were considered as separate data, but for neighboring lakes near each other, and hence constituting a functional unit from breeding ducks’ point of view, data from two or more neighboring lakes (group of lakes) were pooled for a given time series. The lakes in the Finnish study regions represent typical lakes in the boreal northern Europe, ranging from oligotrophic lakes surrounded by forest and peat shores to eutrophic lakes surrounded by arable lands. More information about the Finnish study regions and lakes is given in Heath and Evans (2000, p. 252; Hollola), Pöysä (2001; Parikkala), Kauppinen (1993; Kuopio), and Väänänen (2001; Maaninka).

For each species, we included only complete time series in which zero counts (i.e., no breeding pairs observed in censuses in a given year) occurred in fewer than half of the total years surveyed.

### 2.2 Population variability

For descriptive purposes, we examined overall population variability using the population variability measure (PV; range 0–1) introduced by Heath (2006). This measure quantifies variability among all combinations (years) of observed abundances; PV = 0 means complete stability among years, while a value of PV = 1 is approached as differences in population size approach infinity (for further details, see Heath, 2006).

### 2.3 Bayesian modeling of population processes

To model population dynamics on a site (i.e., community) level, we used hierarchical state‐space formulation on multispecies datasets. Multispecies perspective was used as it allowed the estimation of positive or negative interactions of different species in responses to environmental fluctuations (Mutshinda et al., 2011). We note that, even though we used a multispecies modeling approach, interspecific interactions were not considered in the models; using a similar modeling approach, Almaraz, Green, Aguilera, Rendon, and Bustamante (2012) found that interspecific interactions explained only a negligible proportion of population variances of individual species in a waterfowl community in the Guadalquivir Marshes, southwest Spain. The authors concluded that there was no support for the inclusion of any interspecific effect in the stochastic community dynamics model developed by them. For an underlying population dynamical process, we assumed a Gompertz model, which has been widely applied in modeling studies of various animal populations (Almaraz et al., 2012; Dennis et al., 2006; Mutshinda, O'Hara, & Woiwod, 2009; Mutshinda et al., 2011). The following model description is based on the notation of Mutshinda et al. (2011). Let *N*_*i,t*_ indicate the state number of individuals of species *i* in a community at year *t*, and then, the assumed dynamics becomes the following:

(1) $$N_{i,t}=N_{i,t-1}\exp\left\{r_{i}\left(\frac{1-\log N_{i,t-1}}{k_{i}}\right)+\varepsilon_{i,t}\right\}$$

where *r*_*i*_ is the intrinsic growth rate, and *k*_*i*_ denotes the natural logarithm of carrying capacity of species *i*; ε_*i,t*_ is assumed to be a random process of errors with overall mean of zero and variance determined by demographic stochasticity and environmental variability. On a logarithmic scale, equation (1) becomes the following:

(2) $$n_{i,t}=n_{i,t-1}+r_{i}\left(\frac{1-n_{i,t-1}}{k_{i}}\right)+\varepsilon_{i,t}$$

where *n*_*i,t*_ is the natural logarithm of *N*_*i,t*_. In the matrix form of equation (2), $\varepsilon_{t}={\left(\varepsilon_{1,t},\;\varepsilon_{2,t},\ldots ,\;\varepsilon_{S,t}\right)}^{T}$ is the vector of errors for each species (1, 2,*…*,*S*) in year *t*, assumed to be a multivariate normal distribution (MVN) with mean a vector of zeros and covariance matrix denoted as **Σ**_*t*_; that is, $\varepsilon_{t}=\text{MVN}\left(0,\;\Sigma_{t}\right)$. The covariance matrix **Σ**_*t*_ can be further divided into demographic and environmental variance components:

(3) $$\Sigma_{t}=D_{t}+C$$

where ***C*** is the environmental covariance matrix, in which elements on the main diagonal (*C*_*i,i*_) correspond to species‐specific responses to latent (unspecified) environmental variation (hereafter environmental variability) and off‐diagonal elements (*C*_*i,j*_, *i* ≠ *j*) denote the corresponding joint responses of different species; between‐species covariances with respect to environmental variability were taken from these covariance matrices. The demographic variances of each species in a community were set to be inversely related to the state population sizes indicated by the main diagonal of the matrix, $\text{diag}\left(D_{t}\right)=\delta_{i}^{2}/N_{i,t-1}$, the off‐diagonal elements being zeros. ***D***_*t*_ accounts for a population‐level demographic stochasticity effect on species *i* from year *t* − 1 to year *t*.

Intraspecific interaction *I*_*i*_ (hereafter density dependence) for species *i* is denoted as follows:

(4) $$I_{i}={\left(\frac{r_{i}}{k_{i}}\right)}^{2}\text{Var}\left(n_{i}\right),$$

where Var(**n**_*i*_) is temporal process variance for species *i*. Total variance due to density dependence and environmental variability for species *i* is then: *I*_*i*_ + *C*_*i,i*_. For instance, the proportion of the population dynamics of a species *i* attributed to density dependence is as follows:

(5) $${\text{Prop}}_{\left(I_{i}\right)}=\frac{I_{i}}{I_{i}+C_{i,i}}.$$

The species‐specific observation model was specified with Gaussian errors. Let *Y*_*i,t*_ represent the observed count of species *i* in year *t* in a community, including measurement error. Taking the natural logarithm, *y*_*i,t*_ = log(*Y*_*i,t*_), let us assume the following relation:

(6) $$y_{i,t}|n_{i,t}∼\text{Normal}\left(n_{i,t},\tau_{i}^{2}\right),$$

Bayesian models require explicit priors for all unknown quantities. We set the covariance matrix ***C*** to be the inverse of Wishart(df,Ω) prior, in which *df* is degrees of freedom, that is, the number of species in a given community, and **Ω** is a *df*‐dimensioned identity matrix. Priors for the rest of the model parameters were determined as follows: *r*_*i*_ ∼ Normal(0, 1)*B*(0, ∞), in which *B* is diffuse boundary function, generating posterior comprising of the upper 50% of the normal distribution (values > 0); $k_{i}∼\text{Uniform}\left(k_{\min i},\;k_{\max i}\right)$, where $k_{\min\;i}=\text{Mean}\left(n_{i}\right)-2.576\cdot SD\left(n_{i}\right)$ and $k_{\max i}=\text{Mean}\left(n_{i}\right)+2.576\cdot SD\left(n_{i}\right)$; Uniform (0, 10) was used for standard deviations τ_*i*_ and δ_*i*_ (for uniform noninformative priors, see Gelman, 2006; Kéry & Schaub, 2012).

In order to sample from the joint posterior of the model parameters, we used Markov chain Monte Carlo (MCMC) simulations (Gilks, Richardson, & Spiegelhalter, 1996) implemented with OpenBUGS version 3.2.3 (Thomas, O'Hara, Ligges, & Sturtz, 2006). We used R version 3.1.1 (R Core Team 2014) and package R2OpenBUGS version 3.2‐2.2 (Sturtz, Ligges, & Gelman, 2005) for the preparation of data as well as running and summarizing the simulations. For each model parameter, we initialized four simulation chains and ran 20,000 iterations, discarding the first 10,000 samples of each chain as burn‐in. Markov chains were thinned to every 20th iteration. Convergence of the MCMC simulations was good for each parameter as indicated by the low $\hat{R}$ values (i.e., <1.1; Gelman et al., 2003; see Tables 1 and 2; see also Table S2).

**Table 1:** Continent effect coefficients based on second Bayes (see Methods) for the separate contributions of environmental variability (*C*_*i,i*_) and density dependence (*I*_*i*_) to population dynamics in duck population counts and for the proportion of density dependence (Prop(*I*_*i*_)) of the total variance explained by environmental variability and density dependence of population dynamics

Parameter	Continent effect coefficient		$\hat{R}$	*p*
	Mean	*SD*		
*C*_*i,i*_	0.070	0.034	1.0010	.979
*I*_*i*_	−0.010	0.017	1.0012	.738
Prop(*I*_*i*_)	−0.088	0.065	1.0019	.934

Mean and standard deviation of the coefficients are given as well as $\hat{R}$ values describing the convergence of the MCMC simulations. Rightmost column (*p*) gives probabilities that the coefficient deviates from zero; probabilities were derived from the posterior distribution of each coefficient.

**Table 2:** Guild effect coefficients based on second Bayes (see Methods) for the separate contributions of environmental variability (*C*_*i,i*_) and density dependence (*I*_*i*_) to population dynamics in duck population counts and for the proportion of density dependence (Prop(*I*_*i*_)) of the total variance explained by environmental variability and density dependence of population dynamics

Parameter	Guild effect coefficient		$\hat{R}$	*p*
	Mean	*SD*		
Europe				
*C*_*i,i*_	0.034	0.022	1.0025	.948
*I*_*i*_	−0.003	0.007	1.0021	.661
Prop(*I*_*i*_)	−0.068	0.034	1.0003	.980
North America				
*C*_*i,i*_	0.098	0.095	1.0005	.855
*I*_*i*_	0.015	0.026	1.0017	.710
Prop(*I*_*i*_)	0.062	0.067	1.0021	.828

Mean and standard deviation of the coefficients are given as well as $\hat{R}$ values describing the convergence of the MCMC simulations. Rightmost column (*p*) gives probabilities that the coefficient deviates from zero; probabilities were derived from the posterior distribution of each coefficient.

Bayesian analyses as described above were performed separately for each community; below these are called first Bayes, the posteriors of which form the basis of subsequent Bayesian analyses (next section); second Bayes were performed in order to compare population parameters between continents as well as guilds.

In order to reveal the distribution of continent effect coefficient to species‐ or species pair‐specific population dynamical parameters, we designed second Bayes model based on first Bayes (above) posterior means and standard deviations. A similar procedure was followed for comparisons of guild effect coefficients separately for European and North American duck population time series. We used Bayesian mixed model formulated as follows:

(7) $$\pi_{x}=\alpha_{z}+\beta c_{x}+\varepsilon_{x}$$

where π_*x*_ is second Bayes estimate representing one of the parameters *C*_*i,i*_, *I*_*i*_, or Prop(*I*_*i*_) (eqs. (3), (4), (5); see also Tables 1 and 2), the parameters being specific to each species (*i*) and community. Each community and species combination is indicated by subscript *x*. Parameter α_*z*_ is random term representing variation between communities, subscript *z* indicating community. The random term is specified as $\alpha_{z}∼\text{Normal}\left(\mu ,\;\sigma_{\alpha }^{2}\right)$ with noninformative priors μ∼Normal(0,10,000) and $\sigma_{\alpha }∼\text{Uniform}\left(0,\;10\right)$. Parameter β accounts for the effect of continent indicated by dummy variable *c*_*x*_ (0 = Europe, 1 = North America); ε_*x*_ is residual error. Noninformative priors were set as follows: β∼Normal(0,10,000) and $\varepsilon_{x}∼\text{Normal}\left(0,\;\sigma_{\varepsilon }^{2}\right)$, where $\sigma_{\varepsilon }∼\text{Uniform}\left(0,\;10\right)$. The structure of equation (7) is mixed model that includes both random and fixed effects (Ke'ry & Schaub, 2012; Zuur, Ieno, Walker, Saveliev, & Smith, 2009).

The observation model is written as follows:

$$p_{x}∼\text{Normal}\left(\pi_{x},\sigma_{p,x}^{2}\right)$$

where *p*_*x*_ is the posterior mean (for each *x*) of inspected population parameter taken from first Bayes; standard deviations σ_*p*,*x*_ are informative priors based on corresponding first Bayes posteriors (for informative priors, see Kéry & Royle, 2016; McCarthy & Masters, 2005). The comparison of guilds based on datasets from Europe was similar as explained above (for eq. (7)), but *c*_*x*_ was replaced by dummy variable indicating different guilds (0 = dabbling, 1 = diving), and of course, terms for continent effect were omitted. In North American datasets, there were only two communities. Thus, random term was changed to a factorial dummy variable, and an intercept term was included with respective parameters defined similarly as β above (eq. (7)).

Second Bayes was performed with R package R2jags running program JAGS version 3.4.0 (Plummer, 2003). In second Bayes, four simulation chains were initialized and 20,000 iterations were run, and the first 10,000 samples of each chain were discarded as burn‐in. Markov chain thinning was set to retain every 20th iteration. For the step‐by‐step specification of procedures and the structure of all models, see Table S3.

## 3 Results

Population variability appeared to be lower in dabbling ducks than in diving ducks but comparable between continents for both guilds (dabbling ducks; Europe, 0.417 ± 0.021, *n* = 41; North America, 0.442 ± 0.030, *n* = 12; diving ducks; Europe, 0.576 ± 0.028, *n* = 15; North America, 0.569 ± 0.070, *n* = 4).

**Figure 2:**
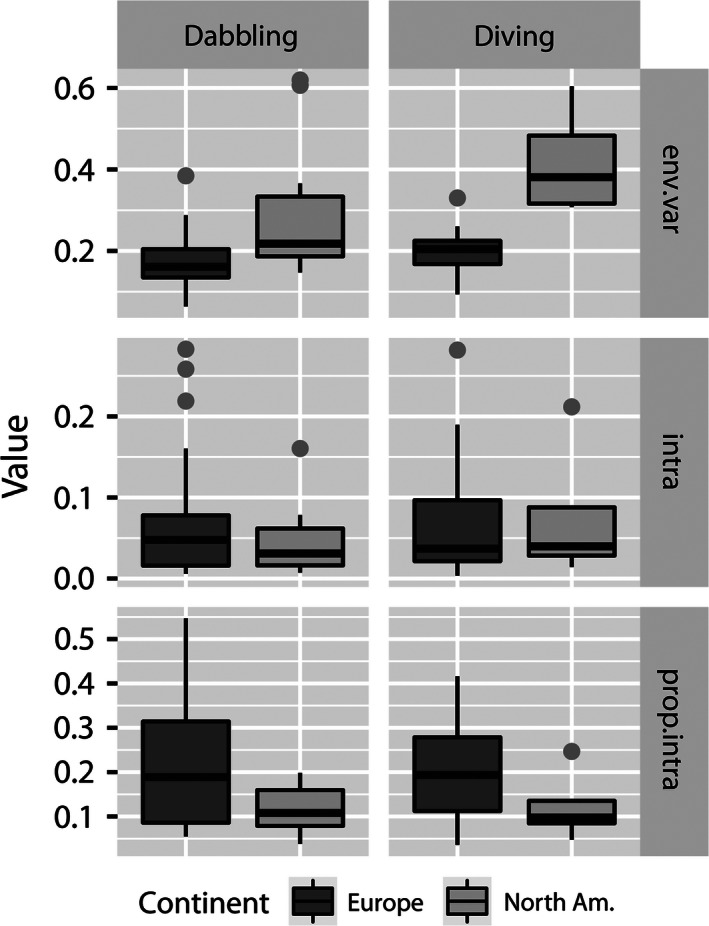
The separate contributions of environmental variability (*C*_*i,i*_; env.var) and density dependence (*I*_*i*_; intra) to population dynamics and the proportion of density dependence of the total variance explained by density dependence and environmental variability of population dynamics (i.e., *I*_*i*_/(*I*_*i*_ + *C*_*i,i*_); prop.intra) in dabbling duck (Dabbling) and diving duck (Diving) population time series for northern Europe (Europe) and North American prairies (North Am.). The upper whisker extends to the highest value that is within 1.5 × IQR, where IQR is the interquartile range, or distance between the first and third quartiles, as indicated by the hinge. The lower whisker extends to the lowest value within 1.5 × IQR. Data beyond the whisker ends are outliers and plotted as points. Data points express species‐ and site‐specific values

**Figure 3:**
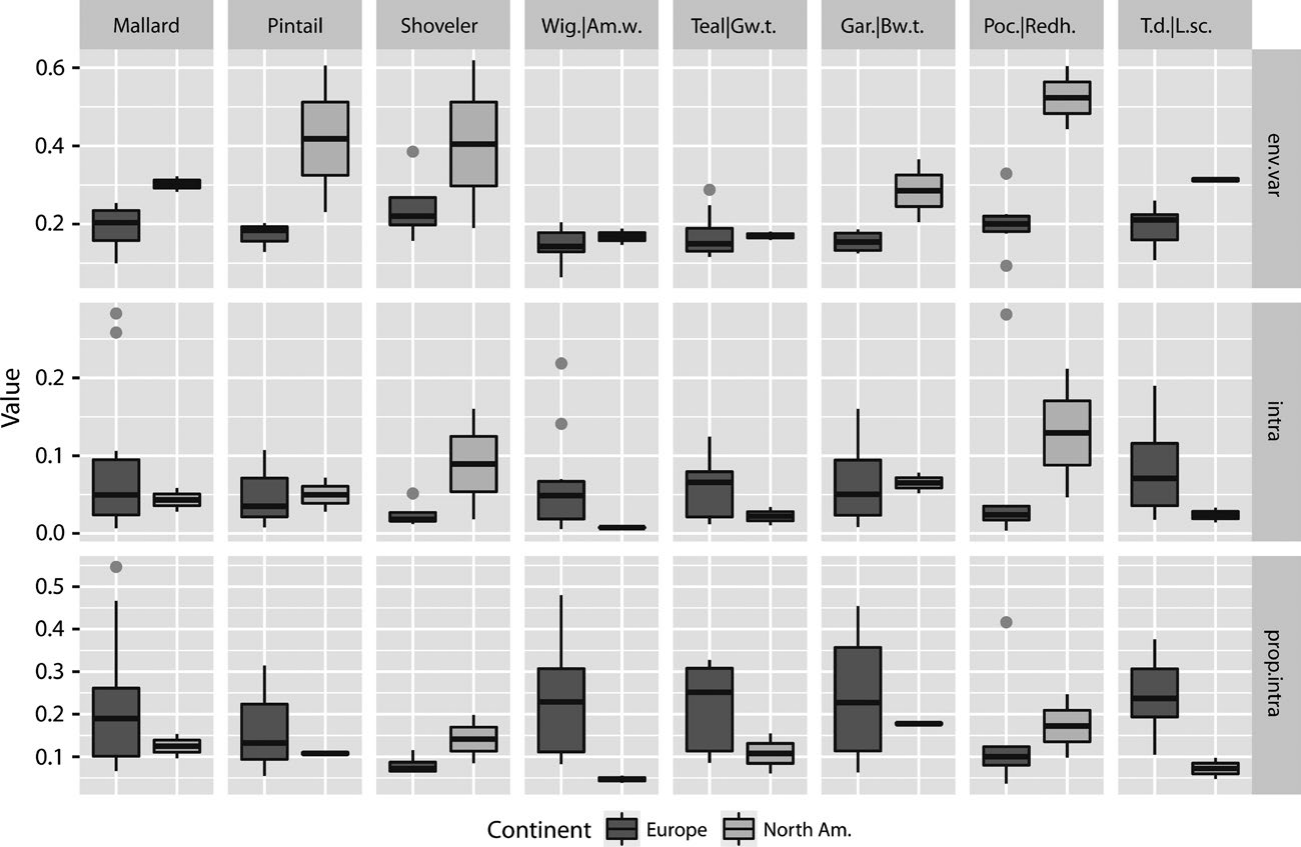
The separate contributions of environmental variability (*C*_*i,i*_; env.var) and density dependence (*I*_*i*_; intra) to population dynamics and the proportion of density dependence of the total variance explained by density dependence and environmental variability of population dynamics (i.e., *I*_*i*_/(*I*_*i*_ + *C*_*i,i*_); prop.intra) in six species pairs of dabbling ducks (first six panels from the left) and two species pairs of diving ducks (last two panels on the right) for northern Europe (Europe) and North American prairies (North Am.). Same species or matched species [close ecological counterparts] from Europe and North America included (for species, see Methods). The upper whisker extends to the highest value that is within 1.5 × IQR, where IQR is the interquartile range, or distance between the first and third quartiles, as indicated by the hinge. The lower whisker extends to the lowest value within 1.5 × IQR. Data beyond the whisker ends are outliers and plotted as points. Data points express species‐ and site‐specific values

All in all, the contribution of environmental variability (*C*_*i,i*_) to population dynamics was greater than that of density dependence and also greater in North America than in Europe (Fig. 2, Table 1). In addition, between‐species (species pairs) covariances in environmental variability within communities were greater in North American than in Europe (see Fig. S1). The contribution of density dependence (*I*_*i*_) to population dynamics was only somewhat greater in Europe than in North America (Table 1), although it was generally low (Fig. 2; see also Fig. 3). As a consequence, the proportion of variation due to density dependence in population dynamics (Prop(*I*_*i*_)) was less in North America than in Europe (Fig. 2, Table 1); species‐ and site‐specific posterior means ranged from 3.8% to 24.5% in North America and from 3.6% to 54.7% in Europe (Figs 2 and 3, “prop.intra”).

The contribution of environmental variability to population dynamics was greater in diving ducks than in dabbling ducks in both Europe and North America, but the difference was not clear in the latter continent (Figs 2 and 3, Table 2). By contrast, differences between dabbling ducks and diving ducks in the contribution of density dependence were not clear in the European data, nor were they particularly clear in the North American time series (Figs 2 and 3, Table 2). However, the proportion of variation due to density dependence in population dynamics was greater in dabbling ducks than in diving ducks in Europe.

Demographic stochasticity appeared to be similar in the European and North American duck communities (see Fig. S2).

## 4 Discussion

Our main findings can be summarized as follows. First, contrary to expectations, populations in the more stable environments of northern Europe did not show lower variability (PV) and showed only slightly more density dependence‐driven dynamics than populations living in the highly variable environments of North American prairies. Second, the contribution of environmental variability to population dynamics was higher in North America than in Europe, a finding in line with expectations concerning the overall importance of environmental stochasticity in population dynamics. Third, populations of presumed “slow species” (diving ducks) were less stable and did not show more density dependent‐driven dynamics than populations of presumed “fast species” (dabbling ducks). As a corollary, the contribution of environmental variability to population dynamics was higher, and hence, the proportion of variation due to density dependence in population dynamics was lower, in “slow species” than in “fast species” in Europe. These latter findings in principle contradict our second prediction concerning the importance of life history in mediating population dynamical responses to environmental variation.

We restricted analyses to duck counts by researchers on the ground, yielding studies from two sites in North America and four in Europe (Finland). This invites criticism that they might not be representative of duck dynamics for these two guilds and thus whether our findings can be generalized to additional local populations over large spatial scales. In the North American prairies, the patterns of population dynamics are representative of the larger prairie biome, especially compared to regions where wetland numbers fluctuate less, as confirmed by many studies of duck population dynamics using data collected during aerial surveys over very wide regions (Bethke & Nudds, 1995; Drever, 2006; Sæther et al., 2008). Corresponding large‐scale data and analyses are not available from Europe. However, data from the Finnish waterfowl monitoring program, extending over the entire country (see Pöysä, Rintala, Lehikoinen, & Väisänen, 2013), make it possible to assess the representativeness of the Finnish study sites at the national scale. To do that, we correlated the annual site‐specific pair numbers (pooled within each of the four sites of this study) of each species with the annual abundance indices from the national monitoring program (both datasets from 1986–2009). We found that, in general, between‐year variation in the site‐ and species‐specific time series correlated well with that in the national abundance indices (mean correlation coefficient, *r* = .466, range −.170 to .810, *n* = 28 site‐ and species‐specific time series). Furthermore, because the ducks breeding in Finland encompass a high proportion of the total European population for many of the species studied here (see Hagemeijer & Blair, 1997), the population dynamics of Finnish ducks should reflect reasonably well the dynamics of ducks breeding in stable European lakes. Thus, we are comfortable that the long‐term data from these sites are representative and that the implication that our inferences generalize to other local populations across broad geographic scales both in North America and in northern Europe.

Our study did not provide support for the idea that low population variability is associated with strong density‐dependent regulation; populations of dabbling ducks appeared to be less variable, but did not show stronger density dependence. Comparisons with earlier studies should be carried out with caution because analysis methods have changed, especially in estimating density dependence (see Koons, Gunnarsson, Schmutz, & Rotella, 2014; Lebreton & Gimenez, 2013; Ross et al., 2015; Roy et al., 2016). Nevertheless, Holyoak and Baillie (1996a) found, among British birds, that annual variability and the strength of density dependence were negatively correlated, a pattern they attributed to variation in longevity; that is, short‐lived species tend to have greater annual population variability than long‐lived species. Similarly, Sæther, Engen, and Matthysen (2002), Sæther, Grøtan, Engen, Noble, and Freckleton (2011) found that species with larger clutch size and lower survival tended to have more variable populations than low‐producing long‐lived species. The species studied by Holyoak and Baillie (1996a) were taxonomically diverse and varied considerably in terms of life history and body size, ranging from 5–6 to 500–600 g (see Holyoak & Baillie, 1996b); species included in Sæther et al. (2002, 2011) varied even more. The ducks studied by us constitute a much more homogeneous group, female body masses ranging approximately from 280 to 1,050 g in dabbling ducks and from 680 to 910 g in diving ducks (Rohwer, 1988). Finally, Williams et al. (2003) did not find a clear connection between population variability and the strength of density dependence in three game birds species in Kansas, USA. All in all, our results and previous findings together suggest that there may not be a general rule about the association between population variability and density‐dependent regulation in birds (see also Sæther et al., 2016).

Our results are generally contrary to conventional wisdom about the relation between life history and characteristics of population dynamics in North American ducks (see Introduction). The original hypothesis was based on life history features, describing the stability of habitats used by each species and the correlation between species density and pond density (see Johnson & Grier, 1988). A key feature of particular interest in the present context is the idea that the settling pattern of a species is assumed to be related to the stability of the habitat it selects. Species living in variable environments will exhibit opportunistic responses to habitat change (i.e., “fast species”), whereas species living in more stable environments will exhibit strong homing tendency and be less responsive (i.e., “slow species”). Hence, populations of the former type are expected to be influenced more by density‐independent factors and populations of the latter type more by density‐dependent factors (Johnson & Grier, 1988). Results from several earlier studies, discussed in Johnson and Grier (1988), provide some support for this hypothesis (Bailey, 1981; Patterson, 1979; Vickery & Nudds, 1984), while more recent studies do not (Lawrence et al., 2013; Murray et al., 2010). Nevertheless, considering the dramatic difference in environmental variability between the European and North American study areas (Fig. 1), our results were surprising in that European duck populations, in general, were not more stable, nor more strongly governed by density dependence, than North American populations. Equally surprising, in northern Europe, populations of species generally considered “slow” (diving ducks) were not more stable and did not exhibit more density dependent‐driven dynamics than populations of presumed “fast” species (dabbling ducks). Information on homing rate in European ducks is limited, but extensive capture–recapture and band recovery data from one wetland area in Latvia suggest that breeding females of the two diving duck species included in our analyses (i.e., Pochard and Tufted Duck) have very high fidelity rates, as does the Northern Shoveler, a dabbling duck (Blums, Nichols, Hines, & Mednis, 2002). Low demographic stochasticity in both continents (Fig. S2) also suggests high fidelity rates. Low homing rate may thus not explain the relatively high population variability and weak contribution of density dependence observed in the European diving ducks.

Our finding that population counts of ducks did not indicate particularly high importance of density dependence corroborates the findings of Lawrence et al. (2013) and Sæther et al. (2008), who analyzed large‐scale species abundance data from North American breeding duck surveys (see also Roy et al., 2016). Weak density dependence is somewhat unexpected, however, because there are both experimental and observational evidences of density dependence in crucial demographic parameters, especially for European dabbling ducks (review in Gunnarsson et al., 2013). On the other hand, little is known about density dependence of vital rates in the diving duck species studied here, and information about density dependence of vital rates for North America ducks in general is scant (see Gunnarsson et al., 2013). Interestingly, using demographic and population data from 13 bird species other than ducks, Sæther et al. (2016) found that, even though density dependence in survival influenced population regulation, environmental stochasticity rather than variation in the strength of density dependence was the major factor affecting interspecific differences in population variability. At any rate, our results suggest that earlier findings from North American ducks may not be generally applicable, at least not to European ducks. Because dabbling ducks and diving ducks breed in the same stable environments in northern Europe, environmental variability, as measured as between‐year variability in wetland numbers, cannot be the primary driving factor for the difference in population variability between the guilds. We focused on only one aspect of environmental variability and on breeding grounds, although the characteristics addressed arguably are crucial for ducks. Obviously, there are other influential and variable exogenous factors such as, for instance, conditions on winter grounds (e.g., Kauppinen & Väänänen, 1999; Pöysä & Väänänen, 2014), whose role in affecting duck population variability and dynamics should be addressed in future studies (see also Koons et al., 2014). In addition, due to demographic stochasticity, populations may fluctuate considerably even in a seemingly constant environment, especially at small population sizes like those in the present study (see Table S1). However, the contribution of demographic stochasticity to population dynamics was estimated to be relatively weak and of similar magnitude in Europe and North America.

In general, irrespective of continent and guild, environmental variability was more important than density dependence in driving population dynamics of ducks, accounting for between ca. 45% and 95% of the total variance explained by these two variance components. The effect of environmental variability on population dynamics depends on the temporal structure of the variation and the responsiveness of the species to it (Roughgarden, 1975; Ripa & Heino, 1999; Schwager et al., 2006). In general, population variability should increase with responsiveness of the species. However, if the environmental variability is highly predictable, the influence of responsiveness is reduced; that is, both “responsive” and “sluggish” species (sensu Roughgarden, 1975) are able to track the variation in the environment. On the other hand, in an unpredictable environment, population dynamics of a species is governed mainly by its responsiveness to environmental variation (see Roughgarden, 1975). The breeding environments of the North American ducks studied here exhibit variability that is worth discussing in light of these ideas. The correlation in the number of May ponds between consecutive years was weak in Johnson's (1995) study area (see Fig. 1; autocorrelation with a lag of 1 year, *r* = −.005), indicating unpredictable environment on 1‐year time scale (i.e., “white noise,” e.g., Roughgarden, 1975; Ruokolainen, Lindén, Kaitala, & Fowler, 2009). Johnson (1995) found that the number of breeding pairs of ducks in the study area was positively correlated with May ponds in the same year for all of the eight species included here, but significantly so for only Blue‐winged Teal and Northern Shoveler. Leitch and Kaminski (1985) found in their study area in Saskatchewan, Canada, that the corresponding correlation was significant for all the species included here, except the Mallard, Northern Shoveler, and Northern Pintail. On the contrary, Sæther et al. (2008) found that the contribution of the temporal variation in pond numbers to annual changes in population size of North American prairie ducks was generally small, except for the Mallard. On the other hand, these authors also found that population variability decreased with decreasing environmental variability, due to lessening fluctuation in pond numbers, in four of the six dabbling duck species studied; these findings were associated with latitudinal gradients in environmental covariates and duck population dynamics in a complex way (see Sæther et al., 2008 for a comprehensive discussion; see also Feldman et al., 2015). In sum, current knowledge of the responsiveness of different species to the annual variation in pond numbers does not allow generalizations about differences between dabbling and diving ducks in population dynamics. Moreover, in addition to the direct response of breeding numbers to variation in pond numbers, demographic responses also are to be expected, and their role in driving population dynamics may differ between prairie‐nesting dabbling and diving ducks (e.g., see discussion in Péron et al., 2012).

In conclusion, we compared basic population dynamic characteristics of the same species or close ecological counterparts between two systems differing drastically in variability of an environmental factor recognized to be influential in earlier studies of duck populations in one of the systems. We found that basic dynamics (temporal variation in population size and density dependence) of the populations in the system in which that particular environmental variability is absent was indistinguishable from the dynamics of the populations in the highly variable system. Furthermore, irrespective of continent and guild, environmental stochasticity was more important than density dependence in driving population dynamics of ducks. These findings lead to a more general conclusion, joining the suggestion by Krebs (2002), that we need more data and studies on populations of the same species in different environments to verify the generality of our explanations about population dynamics.

## Conflict of Interest

None declared.

## Funding Information

None declared.

## References

[ece32413-bib-0001] Almaraz, P. , Green, A. J. , Aguilera, E. , Rendon, M. A. , & Bustamante, J. (2012). Estimating partial observability and nonlinear climate effects on stochastic community dynamics of migratory waterfowl. Journal of Animal Ecology, 81, 1113–1125. 22372885 10.1111/j.1365-2656.2012.01972.x

[ece32413-bib-0002] Bailey, R. O. (1981). A theoretical approach to problems in waterfowl management. Transactions of the North American Wildlife and Natural Resources Conference, 46, 58–71.

[ece32413-bib-0003] Batt, B. D. J. , Anderson, M. G. , Anderson, C. D. , & Caswell, F. D. (1989). The use of prairie potholes by North American ducks In van der Valk A. (Ed.), Northern Prairie wetlands (pp. 204–227). Ames, IA: Iowa State University Press.

[ece32413-bib-0004] Benton, T. G. , Lapsley, C. T. , & Beckerman, A. P. (2002). The population response to environmental noise: Population size, variance and correlation in an experimental system. Journal of Animal Ecology, 71, 320–332.

[ece32413-bib-0005] Bethke, R. W. (1993). Geographical patterns of persistence in duck guilds. Oecologia, 93, 102–108. 28313781 10.1007/BF00321198

[ece32413-bib-0006] Bethke, R. W. , & Nudds, T. D. (1995). Effects of climate change and land use on duck abundance in Canadian prairie‐parklands. Ecological Applications, 5, 588–600.

[ece32413-bib-0007] Bjørnstad, O. N. , & Grenleff, B. T. (2001). Noisy clockwork: Time series analysis of population fluctuations in animals. Science, 293, 638–643. 11474099 10.1126/science.1062226

[ece32413-bib-0008] Blums, P. , Nichols, J. D. , Hines, J. E. , & Mednis, A. (2002). Sources of variation in survival and breeding site fidelity in three species of European ducks. Journal of Animal Ecology, 71, 438–450.

[ece32413-bib-0009] Bonenfant, C. , Gaillard, J.‐M. , Coulson, T. , Festa‐Bianchet, M. , Loison, A. , Garel, M. , … Duncan, P. (2009). Empirical evidence of density‐dependence in populations of large herbivores. Advances in Ecological Research, 49, 313–356.

[ece32413-bib-0010] Brook, B. W. , & Bradshaw, C. J. A. (2006). Strength of evidence for density dependence in abundance time series of 1198 species. Ecology, 87, 1445–1451. 16869419 10.1890/0012-9658(2006)87[1445:soefdd]2.0.co;2

[ece32413-bib-0011] Dennis, B. , Ponciano, J. M. , Lele, S. R. , Taper, M. L. , & Staples, D. F. (2006). Estimating density dependence, process noise, and observation error. Ecological Monographs, 76, 323–341.

[ece32413-bib-0012] Drever, M. C. (2006). Spatial synchrony of prairie ducks: Roles of wetland abundance, distance, and agricultural cover. Oecologia, 147, 725–733. 16341889 10.1007/s00442-005-0308-9

[ece32413-bib-0013] Feldman, R. E. , Anderson, M. G. , Howerter, D. W. , & Murray, D. L. (2015). Where does environmental stochasticity most influence population dynamics? An assessment along a regional core‐periphery gradient for prairie breeding ducks. Global Ecology and Biogeography, 24, 896–904.

[ece32413-bib-0014] Gaston, K. J. , & McArdle, B. H. (1994). The temporal variability of animal abundances: Measures, methods and patterns. Philosophical Transactions of the Royal Society of London B: Biological Sciences, 345, 335–358.

[ece32413-bib-0015] Gelman, A. (2006). Prior distributions for variance parameters in hierarchical models. Bayesian Analysis, 1, 515–534.

[ece32413-bib-0016] Gelman, A. , Carlin, J. B. , Stern, H. S. , & Rubin, D. B. (2003). Bayesian data analysis. Boca Raton, FL: Chapman & Hall/CRC.

[ece32413-bib-0017] Gilks, W. R. , Richardson, S. , & Spiegelhalter, D. J. (1996). Introducing Markov chain Monte Carlo In Gilks W. R., Richardson S., & Spiegelhalter D. J. (Eds.), Markov chain Monte Carlo in practice (pp. 1–19). London, UK: Chapman & Hall.

[ece32413-bib-0018] Gonzalez, J. , Dűttmann, H. , & Wink, M. (2009). Phylogenetic relationships based on two mitochondrial genes and hybridization patterns in Anatidae. Journal of Zoology, 279, 310–318.

[ece32413-bib-0019] Gunnarsson, G. , Elmberg, J. , Pöysä, H. , Nummi, P. , Sjöberg, K. , Dessborn, L. , & Arzel, C. (2013). Density dependence in breeding ducks: A review of the evidence. European Journal of Wildlife Research, 59, 305–321.

[ece32413-bib-0020] Hagemeijer W. J. M., & Blair M. J. (Eds.) (1997). The EBCC atlas of European breeding birds: Their distribution and abundance. London, UK: T&A D Poyser.

[ece32413-bib-0021] Hanski, I. (1990). Density dependence, regulation and variability in animal populations. Philosophical Transactions of the Royal Society of London B: Biological Sciences, 330, 141–150.

[ece32413-bib-0022] Hanski, I. , & Woiwod, I. P. (1993). Mean‐related stochasticity and population variability. Oikos, 67, 29–39.

[ece32413-bib-0023] Heath, J. P. (2006). Quantifying temporal variability in population abundances. Oikos, 115, 573–581.

[ece32413-bib-0024] Heath, M. , & Evans, M. (2000). Important bird areas in Europe. BirdLife Conservation Series No. 8. Frome and London, UK: Butler & Tanner Ltd.

[ece32413-bib-0025] Herrando‐Pérez, S. , Delean, S. , Brook, B. W. , Cassey, P. , & Bradshaw, C. J. A. (2014). Spatial climate patterns explain negligible variation in strength of compensatory density feedbacks in birds and mammals. PLoS ONE, 9(3), e91536. 24618822 10.1371/journal.pone.0091536 PMC3950218

[ece32413-bib-0026] Hixon, M. A. , Pacala, S. W. , & Sandin, S. A. (2002). Population regulation: Historical context and contemporary challenges of open vs. closed systems. Ecology, 83, 1490–1508.

[ece32413-bib-0027] Holopainen, S. , Nummi, P. , & Pöysä, H. (2014). Breeding in the stable boreal landscape: Lake habitat variability drives brood production in the teal (*Anas crecca*). Freshwater Biology, 59, 2621–2631.

[ece32413-bib-0028] Holyoak, M. , & Baillie, S. R. (1996a). Factors influencing detection of density dependence in British birds. II. Longevity and population variability. Oecologia, 108, 54–63. 28307733 10.1007/BF00333214

[ece32413-bib-0029] Holyoak, M. , & Baillie, S. R. (1996b). Factors influencing detection of density dependence in British birds. I. Population trends. Oecologia, 108, 47–53. 28307732 10.1007/BF00333213

[ece32413-bib-0030] Jamieson, L. E. , & Brooks, S. P. (2004). Density dependence in North American ducks. Animal Biodiversity and Conservation, 27(1), 113–128.

[ece32413-bib-0031] Johnson, D. H. (1995). Waterfowl communities in the Northern Plains In Cody M. L., & Smallwood J. A. (Eds.), Long‐term studies of vertebrate communities (pp. 391–418). San Diego, CA: Academic Press.

[ece32413-bib-0032] Johnson, D. H. , & Grier, J. W. (1988). Determinants of breeding distributions of ducks. Wildlife Monographs, 100, 1–37.

[ece32413-bib-0033] Kaitala, V. , Ylikarjula, J. , Ranta, E. , & Lundberg, P. (1997). Population dynamics and the colour of environmental noise. Proceedings of the Royal Society B, 264, 943–948. 9304115 10.1098/rspb.1997.0130 PMC1688534

[ece32413-bib-0034] Kauppinen, J. (1983). Methods used in the census of breeding ducks in northern Savo (Finland) at the beginning of the breeding season. Finnish Game Research, 40, 49–81.

[ece32413-bib-0035] Kauppinen, J. (1993). Densities and habitat distribution of breeding waterfowl in boreal lakes in Finland. Finnish Game Research, 48, 24–45.

[ece32413-bib-0036] Kauppinen, J. , & Väänänen, V.‐M. (1999). Factors affecting changes in waterfowl populations in eutrophic wetlands in the Finnish lake district. Wildlife Biology, 5, 73–81.

[ece32413-bib-0037] Kéry, M. , & Royle, J. A. (2016). Applied hierarchical modeling in ecology: Analysis of distribution, abundance and species richness in R and BUGS. Amsterdam, the Netherlands: Elsevier.

[ece32413-bib-0038] Kéry, M. , & Schaub, M. (2012). Bayesian population analysis using WinBUGS: A hierarchical perspective. Amsterdam, the Netherlands: Elsevier.

[ece32413-bib-0039] Knape, J. , & de Valpine, P. (2012). Are patterns of density dependence in the Global Population Dynamics Database driven by uncertainty about population abundance? Ecology Letters, 15, 17–23. 22017744 10.1111/j.1461-0248.2011.01702.x

[ece32413-bib-0040] Koons, D. N. , Gunnarsson, G. , Schmutz, J. A. , & Rotella, J. J. (2014). Drivers of waterfowl population dynamics: From teal to swans. Wildfowl Special Issue, 4, 169–191.

[ece32413-bib-0041] Koskimies, P. , & Väisänen, R. A. (1991). Monitoring bird populations. A manual of methods applied in Finland. Helsinki, Finland: Zoological Museum, Finnish Museum of Natural History.

[ece32413-bib-0042] Krebs, C. J. (2002). Two complementary paradigms for analyzing population dynamics. Philosophical Transactions of the Royal Society of London B: Biological Sciences, 357, 1211–1219. 12396513 10.1098/rstb.2002.1122 PMC1693036

[ece32413-bib-0043] Laakso, J. , Löytynoja, K. , & Kaitala, V. (2003). Environmental noise and population dynamics of the ciliated protozoa *Tetrahymena thermophila* in aquatic microcosms. Oikos, 102, 663–671.

[ece32413-bib-0044] Lande, R. (1993). Risks of population extinction from demographic and environmental stochasticity and random catastrophes. The American Naturalist, 142, 911–927. 10.1086/285580 29519140

[ece32413-bib-0045] Lande, R. , Engen, S. , & Sæther, B.‐E. (2003). Stochastic population dynamics in ecology and conservation. Oxford, UK: Oxford University Press.

[ece32413-bib-0046] Lawrence, J. D. , Gramacy, R. B. , Thomas, L. , & Buckland, S. T. (2013). The importance of prior choice in model selection: A density dependence example. Methods in Ecology and Evolution, 4, 25–33.

[ece32413-bib-0047] Lebreton, J.‐D. , & Gimenez, O. (2013). Detecting and estimating density dependence in wildlife populations. Journal of Wildlife Management, 77, 12–23.

[ece32413-bib-0048] Leitch, W. G. , & Kaminski, R. M. (1985). Long‐term wetland‐waterfowl trends in Saskatchewan grassland. Journal of Wildlife Management, 49, 212–222.

[ece32413-bib-0050] McCarthy, M. A. , & Masters, P. (2005). Profiting from prior information in Bayesian analyses of ecological data. Journal of Applied Ecology, 42, 1012–1019.

[ece32413-bib-0051] Murdoch, W. W. (1994). Population regulation in theory and practice. Ecology, 75, 271–287.

[ece32413-bib-0052] Murray, D. L. , Anderson, M. G. , & Steury, T. D. (2010). Temporal shift in density dependence among North American breeding duck populations. Ecology, 91, 571–581. 20392021 10.1890/ms08-1032.1

[ece32413-bib-0053] Mutshinda, C. M. , O'Hara, R. B. , & Woiwod, I. P. (2009). What drives community dynamics? Proceedings of the Royal Society B, 276, 2923–2929. 19457887 10.1098/rspb.2009.0523 PMC2817208

[ece32413-bib-0054] Mutshinda, C. M. , O'Hara, R. B. , & Woiwod, I. P. (2011). A multispecies perspective on ecological impacts of climatic forcing. Journal of Animal Ecology, 80, 101–107. 20809921 10.1111/j.1365-2656.2010.01743.x

[ece32413-bib-0055] Nudds, T. D. (1983). Niche dynamics and organization of waterfowl guilds in variable environments. Ecology, 64, 319–330.

[ece32413-bib-0056] Nudds, T. D. , Sjöberg, K. , & Lundberg, P. (1994). Ecomorphological relationships among Palearctic dabbling ducks on Baltic coastal wetlands and a comparison with the Nearctic. Oikos, 69, 295–303.

[ece32413-bib-0057] Nummi, P. , Holopainen, S. , Rintala, J. , & Pöysä, H. (2015). Mechanisms of density dependence in ducks: Importance of space and per capita food. Oecologia, 177, 679–688. 25398723 10.1007/s00442-014-3133-1

[ece32413-bib-0058] Nummi, P. , & Pöysä, H. (1993). Habitat associations of ducks during different stages of the breeding season. Ecography, 16, 319–328.

[ece32413-bib-0059] Patterson, J. H. (1979). Can ducks be managed by regulation? Experiences in Canada. Transactions of North American Wildlife and Natural Resources Conference, 44, 130–139.

[ece32413-bib-0060] Péron, G. , Nicolai, C. A. , & Koons, D. N. (2012). Demographic response to perturbations: The role of compensatory density dependence in a North American duck under variable harvest regulations and changing habitat. Journal of Animal Ecology, 81, 960–969. 22433018 10.1111/j.1365-2656.2012.01980.x

[ece32413-bib-0061] Petchey, O. L. (2000). Environmental colour affects aspects of single‐species population dynamics. Proceedings of the Royal Society B, 267, 747–754. 10819142 10.1098/rspb.2000.1066 PMC1690600

[ece32413-bib-0062] Pianka, E. R. (1970). On r‐ and K‐selection. The American Naturalist, 104, 592–597.

[ece32413-bib-0063] Plummer, M. (2003). JAGS: A program for analysis of Bayesian graphical models using Gibbs sampling. Retrieved from http://citeseerx.ist.psu.edu/viewdoc/summary?doi=10.1.1.13.3406

[ece32413-bib-0064] Pöysä, H. (1996). Population estimates and the timing of waterfowl censuses. Ornis Fennica, 73, 60–68.

[ece32413-bib-0065] Pöysä, H. (2001). Dynamics of habitat distribution in breeding mallards: Assessing the applicability of current habitat selection models. Oikos, 94, 365–373.

[ece32413-bib-0066] Pöysä, H. , Rintala, J. , Lehikoinen, A. , & Väisänen, R. A. (2013). The importance of hunting pressure, habitat preference and life history for population trends of breeding waterbirds in Finland. European Journal of Wildlife Research, 59, 245–256.

[ece32413-bib-0067] Pöysä, H. , & Väänänen, V.‐M. (2014). Drivers of breeding numbers in a long‐distance migrant, the Garganey (*Anas querquedula*): Effects of climate and hunting pressure. Journal of Ornithology, 155, 679–687.

[ece32413-bib-0068] R Core Team . (2014). R: A languagse and environment for statistical computing. Vienna, Austria: R Foundation for Statistical Computing URL: http://www.R-project.org/.

[ece32413-bib-0069] Ranta, E. , Lundberg, P. , Kaitala, V. , & Laakso, J. (2000). Visibility of the environmental noise modulating population dynamics. Proceedings of the Royal Society B, 267, 1851–1856. 11052535 10.1098/rspb.2000.1220 PMC1690746

[ece32413-bib-0070] Ripa, J. , & Heino, M. (1999). Linear analysis solves two puzzles in population dynamics: The route to extinction and extinction in coloured environments. Ecology Letters, 2, 219–222.

[ece32413-bib-0071] Rohwer, F. C. (1988). Inter‐ and intraspecific relationships between egg size and clutch size in waterfowl. The Auk, 105, 161–176.

[ece32413-bib-0072] Ross, B. E. , Hooten, M. B. , DeVink, J.‐M. , & Koons, D. N. (2015). Combined effects of climate, predation, and density dependence on greater and lesser scaup population dynamics. Ecological Applications, 25, 1606–1617. 26552268 10.1890/14-0582.1

[ece32413-bib-0073] Roughgarden, J. (1975). A simple model for population dynamics in stochastic environments. The American Naturalist, 109, 713–736.

[ece32413-bib-0074] Roy, C. , McIntire, E. J. B. , & Cumming, S. G. (2016). Assessing the spatial variability of density dependence in waterfowl populations. Ecography, in press.

[ece32413-bib-0075] Royama, T. (1992). Analytical population dynamics. New York, NY: Chapman & Hall.

[ece32413-bib-0076] Ruokolainen, L. , Lindén, A. , Kaitala, C. V. , & Fowler, M. S. (2009). Ecological and evolutionary dynamics under coloured environmental variation. Trends in Ecology and Evolution, 24, 555–563. 19699550 10.1016/j.tree.2009.04.009

[ece32413-bib-0077] Sæther, B.‐E. (1987). The influence of body weight on the covariation between reproductive traits in European birds. Oikos, 48, 79–88.

[ece32413-bib-0078] Sæther, B.‐E. , Engen, S. , & Matthysen, E. (2002). Demographic characteristics and population dynamical patterns of solitary birds. Science, 295, 2070–2073. 11896278 10.1126/science.1068766

[ece32413-bib-0079] Sæther, B.‐E. , Grøtan, V. , Engen, S. , Coulson, T. , Grant, P. R. , Visser, M. E. , … Weimerskirch, H. (2016). Demographic routes to variability and regulation in bird populations. Nature Communications, 7, 12001. doi:10.1038/ncomms12001 10.1038/ncomms12001 PMC4917965 27328710

[ece32413-bib-0080] Sæther, B.‐E. , Grøtan, V. , Engen, S. , Noble, D. G. , & Freckleton, R. P. (2011). Rarity, life history and scaling of the dynamics in time and space of British birds. Journal of Animal Ecology, 80, 215–224. 20840608 10.1111/j.1365-2656.2010.01751.x

[ece32413-bib-0081] Sæther, B.‐E. , Lillegård, M. , Grøtan, V. , Drever, M. C. , Engen, S. , Nudds, T. D. , & Podruzny, K. M. (2008). Geographical gradients in the population dynamics of North American prairie ducks. Journal of Animal Ecology, 77, 869–882. 18631261 10.1111/j.1365-2656.2008.01424.x

[ece32413-bib-0082] Schwager, M. , Johst, K. , & Jeltsch, F. (2006). Does red noise increase or decrease extinction risk? Single extreme events versus series of unfavorable conditions. The American Naturalist, 167, 879–888. 10.1086/503609 16615033

[ece32413-bib-0083] Sibly, R. M. , Barker, D. , Denham, M. C. , Hone, J. , & Pagel, M. (2005). On the regulation of populations of mammals, birds, fish, and insects. Science, 309, 607–610. 16040705 10.1126/science.1110760

[ece32413-bib-0084] Sinclair, A. R. E. , & Pech, R. P. (1996). Density dependence, stochasticity, compensation and predator regulation. Oikos, 75, 164–173.

[ece32413-bib-0085] Stenseth, N. C. (1999). Population cycles in voles and lemmings: Density dependence and phase dependence in a stochastic world. Oikos, 87, 427–461.

[ece32413-bib-0086] Sturtz, S. , Ligges, U. , & Gelman, A. (2005). R2WinBUGS : A package for running WinBUGS from R. Journal of Statistical Software, 12, 1–16.

[ece32413-bib-0087] Suhonen, S. , Nummi, P. , & Pöysä, H. (2011). Long term stability of boreal lake habitats and use by breeding ducks. Boreal Environment Research, 16(suppl. B), 71–80.

[ece32413-bib-0088] Thomas, A. , O'Hara, R. B. , Ligges, U. , & Sturtz, S. (2006). Making BUGS open. R News, 6, 12–17.

[ece32413-bib-0089] Turchin, P. (1995). Population regulation: Old arguments and a new synthesis In Cappuccino N., & Price P. W. (Eds.), Population dynamics. New approaches and synthesis (pp. 19–40). San Diego, CA: Academic Press.

[ece32413-bib-0090] Turchin, P. (1999). Population regulation: A synthetic view. Oikos, 84, 153–159.

[ece32413-bib-0091] Turchin, P. , & Taylor, A. D. (1992). Complex dynamics in ecological time series. Ecology, 73, 289–305.

[ece32413-bib-0092] Väänänen, V.‐M. (2001). Hunting disturbance and timing of autumn migration in *Anas* species. Wildlife Biology, 7, 3–9.

[ece32413-bib-0094] Vickery, W. L. , & Nudds, T. D. (1984). Detection of density‐dependent effects in annual duck censuses. Ecology, 65, 96–104.

[ece32413-bib-0095] Viljugrein, H. , Stenseth, N. C. , Smith, G. W. , & Steinbakk, G. H. (2005). Density dependence in North American ducks. Ecology, 86, 245–254.

[ece32413-bib-0096] Williams, C. K. , Ives, A. R. , & Applegate, R. D. (2003). Population dynamics across geographical ranges: Time‐series analyses of three small game species. Ecology, 84, 2654–2667.

[ece32413-bib-0097] Woiwod, I. P. , & Hanski, I. (1992). Patterns of density dependence in moths and aphids. Journal of Animal Ecology, 61, 619–629.

[ece32413-bib-0098] Zeng, Z. , Nowierski, R. M. , Taper, M. L. , Dennis, B. , & Kemp, W. P. (1998). Complex population dynamics in the real world: Modeling the influence of time‐varying parameters and time lags. Ecology, 79, 2193–2209.

[ece32413-bib-0099] Ziebarth, N. L. , Abbott, K. C. , & Ives, A. R. (2010). Weak population regulation in ecological time series. Ecology Letters, 13, 21–31. 19849710 10.1111/j.1461-0248.2009.01393.x

[ece32413-bib-0100] Zuur, A. F. , Ieno, E. N. , Walker, N. J. , Saveliev, A. A. , & Smith, G. M. (2009). Mixed effects models and extensions in ecology with R. New York, NY: Springer.

