# Supplementary figures and images for: Environmental variability and population dynamics: do European and North American ducks play by the same rules?
Source: Ecol Evol. 2016 Sep 9;6(19):7004–14. doi: 10.1002/ece3.2413 (PMC5513220; doi:10.1002/ece3.2413)

Fig. S1


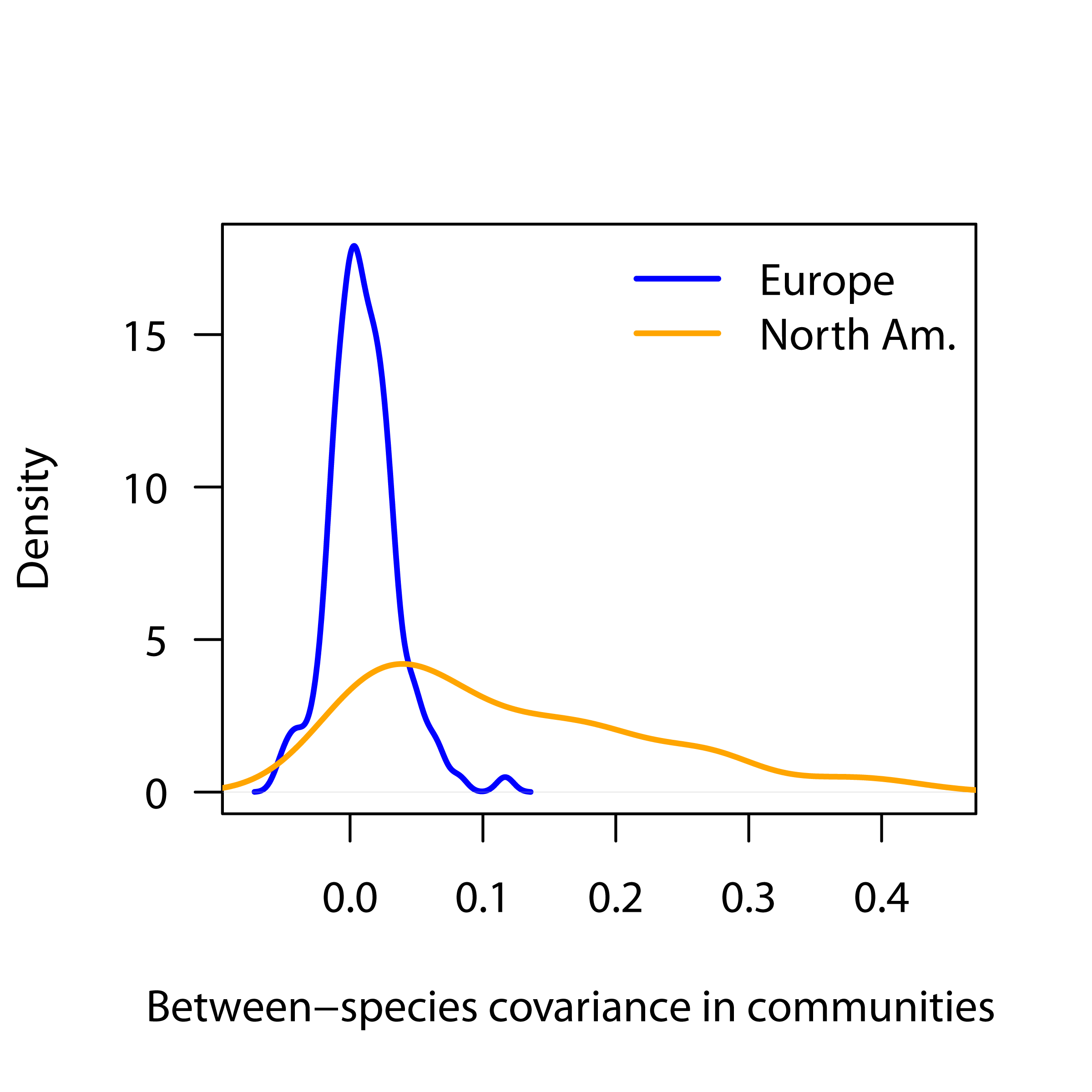

Supplement: Supplementary file 1 [file ECE3-6-7004-s001.doc]

Fig. S2


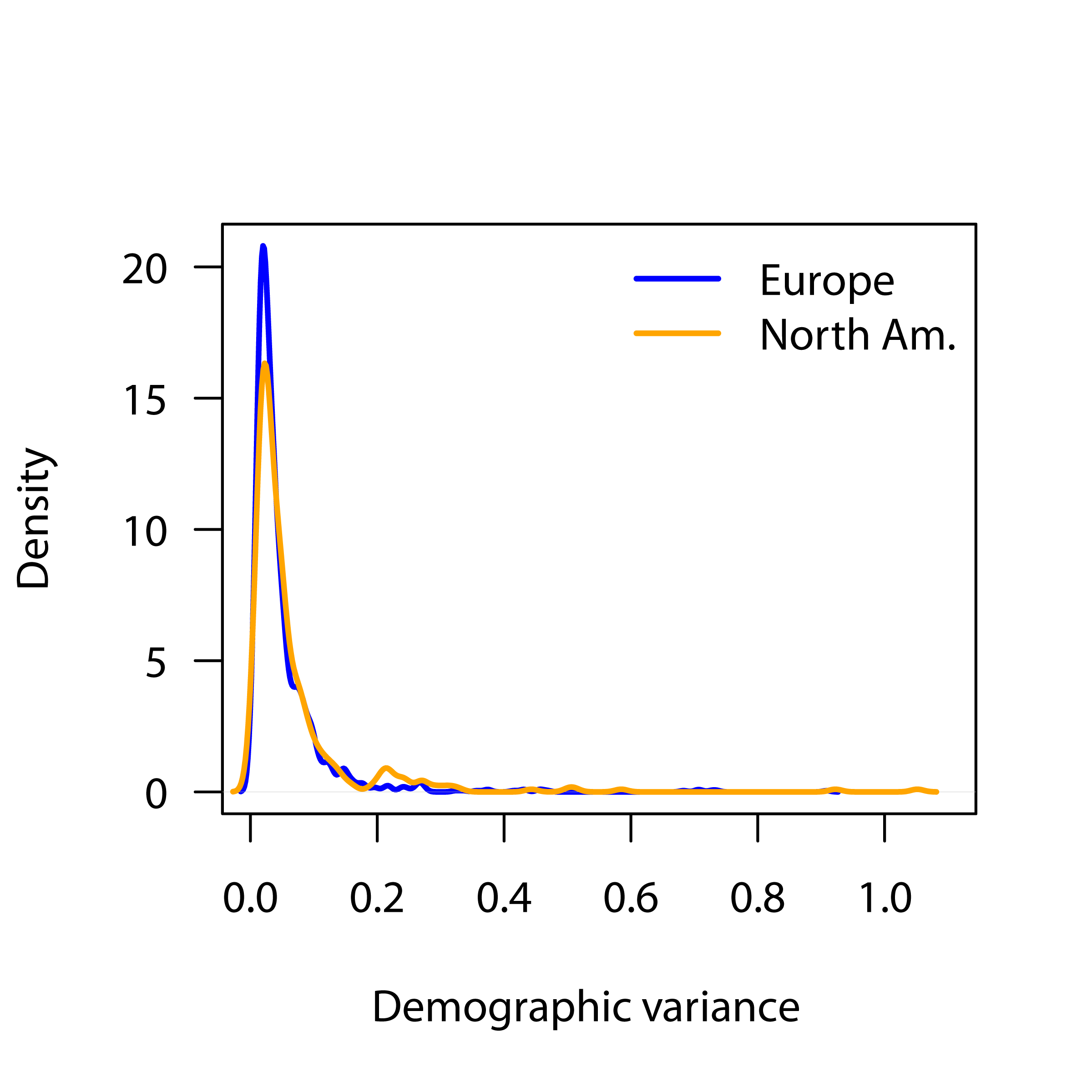

Supplement: Supplementary file 2 [file ECE3-6-7004-s002.doc]
