# Supplementary material for: Environmental variability and population dynamics: do European and North American ducks play by the same rules?
Source: Ecol Evol. 2016 Sep 9;6(19):7004–14. doi: 10.1002/ece3.2413 (PMC5513220; doi:10.1002/ece3.2413)
Supplement: Supplementary file 3 [file ECE3-6-7004-s003.doc]

Table S1. Summary information on the time series of breeding ducks used in the analyses. Entries in the table give the number of time series for each species or matched species for each study area (mean population size in parentheses). For the Hollola and Kuopio study areas there were more than one time series for a given species and they all come from different lakes (or lake groups) and from slightly different study periods (for further information, see main text, Methods, Population data).

Europe North America

Study area: Hollola Parikkala Kuopio Maaninka Redvers, Woodforth,

Saskatchewan North Dakota

Source: Lammi, E. Pöysä, H. Kauppinen, J. Väänänen, V.-M. Vickery and Johnson 1995

(unpublished) (unpublished) (unpublished) (unpublished) Nudds 1983

Study period: 1977-2009 1985-2009 1981-2009 1985-2009 1952-1977 1965-1989

1987-2009 1982-2009

1984-2009

Species

Eurasian Wigeon/

American Wigeon 3 (5.1) 1 (12.2) 5 (4.7) 1 (20.8) 1 (21.6) 1 (9.5)

Mallard/

Mallard 3 (9.2) 1 (21.7) 5 (3.4) 1 (15.4) 1 (82.6) 1 (52.2)

Northern Shoveler/

Northern Shoveler 1 (4.4) 2 (2.0) 1 (30.1) 1 (12.5) 1 (30.3)

Northern Pintail/

Northern Pintail 2 (2.2) 1 (15.2) 1 (14.4) 1 (28.1)

Garganey/

Blue-winged Teal 1 (3.1.) 1 (1.0) 1 (1.5) 1 (9.6) 1 (91.7) 1 (211.2)

Eurasian Teal/

Green-winged Teal 3 (6.5) 1 (15.8) 5 (4.9) 1 (47.9) 1 (17.9) 1 (7.7)

Pochard/

Redhead 2 (7.0) 3 (1.1) 1 (5.2) 1 (4.3.) 1 (25.8)

Tufted Duck/

Lesser Scaup 2 (8.2) 1 (7.4) 5 (3.5) 1 (11.4) 1 (9.8) 1 (29.7)
