# Supplementary material for: Environmental variability and population dynamics: do European and North American ducks play by the same rules?
Source: Ecol Evol. 2016 Sep 9;6(19):7004–14. doi: 10.1002/ece3.2413 (PMC5513220; doi:10.1002/ece3.2413)
Supplement: Supplementary file 5 [file ECE3-6-7004-s005.doc]

Table S3. Step-by-step specification of models and procedures

Environmental variability and population dynamics: do European and North American ducks play by the same rules?

Hannu Pöysä, Jukka Rintala, Douglas H. Johnson, Jukka Kauppinen, Esa Lammi, Thomas D. Nudds and Veli-Matti Väänänen

library(R2OpenBUGS)

library(R2jags)

library(ggplot2)

library(plyr)

library(mgcv)

library(reshape)

library(lattice)

library(Cairo)

library(gridExtra) # multiplot

library(nlme)

rm(list=ls())

#oldwd <- getwd()

setwd("H:\\Data\\am_eu_bugs") # optional

###### 1st Bayes, estimating posteriors for each species and site

w.fun=function(dat2=dat2){

States=NULL

x=1:ncol(dat2)

for(i in 1:nrow(dat2)){

#i=1

y=as.numeric(log(dat2+1)[i,])

#y[is.na(y)] <- mean(y, na.rm=T)

ytmp=y[!is.na(y)]

xtmp=x[!is.na(y)]

b <- gam(ytmp~s(xtmp))

b=predict(b,data.frame(xtmp=x))

b=round(rnorm(length(b), b, .1),3)

States=rbind(States, b)

}

States[,1] <- NA

States=t(States)

States

}

n.fun=function(dat2=dat2){

n=NULL

x=1:ncol(dat2)

for(i in 1:nrow(dat2)){

y=as.numeric(log(dat2+1)[i,])

ytmp=y[!is.na(y)]

xtmp=x[!is.na(y)]

b <- gam(ytmp~s(xtmp))

b=predict(b,data.frame(xtmp=x))

b=round(rnorm(length(b), b, .1),3)

n=rbind(n, b)

}

n=t(n)

n

}

##wishart initials

tau.fun <- function(dat2=dat2){

S <- diag(1, nrow(dat2), nrow(dat2))

R <- rWishart(1, nrow(dat2), S)

Tau=round(R,3)

Tau=matrix(Tau,ncol=nrow(dat2))

Tau

}

##carrying capacity

k.fun <- function(dat2=dat2){

tmpk=apply(log(dat2+1),1,mean)

k=round(rnorm(length(tmpk), tmpk,.05),2)

k

}

##intr. growth

r.fun <- function(dat2=dat2){

r=round(rnorm(nrow(dat2),1,.025),3)

r

}

##demographic stochasticity sd

sdem.fun <- function(dat2=dat2){

sdem=round(runif(nrow(dat2),0.1,5),1)

sdem

}

##Observation error

sds.fun <- function(dat2=dat2){

sds=round(runif(nrow(dat2),0.1,5),1)

sds

}

dat=read.csv2("Pöysä et al. Vesilintuaikasarjat.csv") # read the data

range(log(dat[,2:9]+1), na.rm=T)

melt1=melt(dat, id.vars=c("Vuosi","cont","ref"))

meltCheck=melt(dat, id.vars=c("Vuosi","cont","ref"), na.rm=T)

meltCheck[meltCheck$value==0,"set"] <- "zero"

meltCheck[meltCheck$value>0,"set"] <- "posit"

names(meltCheck) <- c("Vuosi","cont","ref","sp","Count","set")

str(meltCheck)

meltCheck2=melt(meltCheck, id.vars=c("Vuosi","cont","ref","sp","set"))

head(meltCheck2)

Zeros=cast(meltCheck2, cont + ref + variable + sp ~ set, length)

Zeros$prop=Zeros$zero/(Zeros$posit+Zeros$zero)

Zeros[Zeros$prop>0.4,]

tmpref=as.character(Zeros[Zeros$prop>0.4,"ref"])

dat[dat$ref%in%tmpref,]

range(log(melt1$value+1), na.rm=T)

refvec=names(summary(melt1$ ref))

refvec2=refvec[refvec!="Lammi Tiilijärvet aineisto"] # remove the only-one-species site

sim.list=list(vector("list",length(refvec2)),vector("list",length(refvec2)))

length(sim.list)

length(sim.list[[1]])

length(sim.list[[2]])

sink("Am_Eu_vl_model_1.r")

cat("

model {

##### Model incorporating environmental var-cov and demographic stochasticity as well as observation error

Sigma[1:M,1:M]<-inverse(Tau[1:M,1:M])

Tau[1:M, 1:M] ~ dwish(Rho[1:M , 1:M], M)

for(j in 1:M){

r[j] ~ dnorm(0, 1) I(0,) # saved 100000 run

kmin[j]<-mean(n[,j]) - 2.576*sd(n[,j])

kmax[j]<-mean(n[,j]) + 2.576*sd(n[,j])

k[j] ~ dunif(kmin[j],kmax[j])

tau.d[j]<-1/vd[j] # demographic variance from Mutshinda et al. 2011

vd[j]<-sdem[j]*sdem[j]

sdem[j] ~ dunif(0,10)

Samp.prec[j]<-1/var.s[j] # precision

var.s[j]<-sds[j]*sds[j]

sds[j]~dunif(0, 10)

}

############# Sp-to-sp difference of prop.intra (proportion DD)

for(j in 1:(M-1)){

for(i in (j+1):M){

dprop.intra[j,i] <- prop.intra[j]-prop.intra[i]

}

}

############# PROCESS MODEL ###############################################

for(i in 2:numY){

### Incorporating environmental stochasticity #########################

w[i, 1:M]~dmnorm(m[i, 1:M], Tau[,])

for(j in 1:M){

### Tau[,] is the environmental covariance matrix

### Gompertz model ###################

m[i,j]<- n[i-1,j] + r[j]*(1-n[i-1,j]/k[j])

## incorporating demographic stochasticity

Dem.var[i,j]<-1/Dem.prec[i,j]

Dem.prec[i,j]<-tau.d[j]*exp(n[i-1,j])

n[i,j] ~ dnorm(w[i,j], Dem.prec[i,j])

### observation model

DATA[i,j] ~ dnorm(n[i,j],Samp.prec[j])

} # end j

} # end i

for(j in 1:M) {

### initializations #######################################

n[1,j] ~ dnorm(0, 0.01)I(0,)

##DATA[1,j] ~ dnorm(0, 0.01)I(0,)

DATA[1,j] ~ dnorm(n[1,j],Samp.prec[j])

### Total variance for species j #########################

var.n[j] <- pow(sd(n[,j]),2)

totvar[j]<- pow((r[j]/k[j]),2)* var.n[j] + Sigma[j,j]

intra[j] <- pow((r[j]/k[j]),2)* var.n[j]

prop.intra[j] <- intra[j]/totvar[j]

prop.Sigma[j] <- Sigma[j,j]/totvar[j]

env.var[j] <- Sigma[j,j]

} # end model

",fill=TRUE)

sink()

### _________________________ Main Fun_________________________________

main.fun <- function(REF="Johnson aineisto",SEED=1,NITER=500, NTHIN=1, DEBUG=F){

set.seed(SEED)

cast1=cast(melt1, variable+cont+ref~Vuosi, sum, fill=NA, subset=ref==REF)

sums=apply(cast1[,4:ncol(cast1)],1,sum, na.rm=T)

dat2=cast1[sums>0,4:ncol(cast1)]

contvec=cast1[sums>0,"cont"]

refvec=cast1[sums>0,"ref"]

spvec=cast1$ variable[sums>0]

write.csv2(data.frame(nro=1:length(spvec),refvec,spvec),paste("species_",REF,".csv", sep=""))

range(log(dat2+1))

head(data.frame(spvec, refvec,dat2))

data.obj=list(DATA=t(as.matrix(log(dat2+1))),

Rho=diag(nrow(dat2)),

M=nrow(dat2),

numY=ncol(dat2),

combinations=as.matrix(combn(1:ncol(dat2), 2)),

)

inits=list(

list(k=k.fun(dat2),r=r.fun(dat2),sdem=sdem.fun(dat2),sds=sds.fun(dat2),n=n.fun(dat2),w=w.fun(dat2),Tau=tau.fun(dat2)),

list(k=k.fun(dat2),r=r.fun(dat2),sdem=sdem.fun(dat2),sds=sds.fun(dat2),n=n.fun(dat2),w=w.fun(dat2),Tau=tau.fun(dat2)),

list(k=k.fun(dat2),r=r.fun(dat2),sdem=sdem.fun(dat2),sds=sds.fun(dat2),n=n.fun(dat2),w=w.fun(dat2),Tau=tau.fun(dat2)),

list(k=k.fun(dat2),r=r.fun(dat2),sdem=sdem.fun(dat2),sds=sds.fun(dat2),n=n.fun(dat2),w=w.fun(dat2),Tau=tau.fun(dat2)) #4th intits

)

save.para=c("prop.intra","intra","env.var","r","k","Sigma","prop.Sigma","dprop.intra","var.s","Dem.var","n") # ,"w"

sim.result<-bugs(data=data.obj, inits=inits, parameters.to.save=save.para, n.iter=NITER, n.thin=NTHIN, n.burnin=floor(NITER / 2), #

model.file="Am_Eu_vl_model_1.r", n.chains=length(inits), debug=DEBUG)

tmp=print(sim.result)

#tmp$summary

param=row.names(tmp$summary)

param=gsub("[0-9]","",param)

param=gsub(",","",param)

paramInd=row.names(tmp$summary)

tab=data.frame(param=param,tmp$summary,paramInd=paramInd)

SELECT=save.para!="Sigma"&save.para!="dprop.intra"&save.para!="n" &save.para!="Dem.var" # ignore annually varying param, Sigma in filling species names

npar=length(save.para[SELECT]) # ignore w, Sigma

sp=as.character(spvec)

tab[tab$param %in% paste(save.para[SELECT],"[]", sep=""),"sp"] <- rep(sp,npar) # ignore w, Sigma in filling names

names(tab)[2] <- "Mean"

if(REF=="Johnson aineisto"|REF=="Nudds & Vickery aineisto") tab$Continent <- "North Am." else tab$Continent <- "Europe"

Cairo(paste(REF,".png", sep=""), width = 6, height = 6, units="in", dpi=600, pointsize=8)

plot(sim.result)

title(sub=REF, col.sub="blue")

dev.off()

result.list=list(tab,sim.result) # takes a lot of memory

#result.list=list(tab,NULL)

return(result.list)

}

###### __________ end main fun

######################################################################

######################### Run Main Function ##########################

######################################################################

########################## Testing ###

set.seed(1)

seeds=floor(runif(length(refvec2),100000,200000))

sim.list=list(vector("list",length(refvec2)),vector("list",length(refvec2))) # clear

refnum=1

tmp.sim.out <- main.fun(REF=refvec2[refnum], NITER=500, SEED=seeds[refnum],NTHIN=10,DEBUG=F)

sim.list[[1]][[refnum]] <- tmp.sim.out[[1]]

sim.list[[2]][[refnum]] <- tmp.sim.out[[2]]

###################### End Testing ###################################

sim.list=list(vector("list",length(refvec2)),vector("list",length(refvec2))) # clear

rm(tmp.sim.out)

set.seed(1)

seeds=floor(runif(length(refvec2),100000,200000))

systime=vector("list",length(sim.list)+1)

iterations=20000

thinnings=20

debugTF=F

for(refnum in 1:length(refvec2)){ #length(summary(melt1$ ref))

if(refnum==1){

StartTime <- Sys.time()

StartTime

cat("", "\n")

cat("started processing reference :", refnum, refvec2[refnum],paste(Sys.time()), "\n")

tmp.sim.out <- main.fun(REF=refvec2[refnum], NITER=iterations, NTHIN=thinnings, SEED=seeds[refnum], DEBUG=debugTF) #, DEBUG=T

sim.list[[1]][[refnum]] <- tmp.sim.out[[1]]

sim.list[[2]][[refnum]] <- tmp.sim.out[[2]]

tmp.sim.out=NULL

} else {

cat("", "\n")

cat("started processing reference :", refnum, refvec2[refnum],paste(Sys.time()), "\n")

tmp.sim.out <- main.fun(REF=refvec2[refnum], NITER=iterations, SEED=seeds[refnum], DEBUG=debugTF) #,DEBUG=T

sim.list[[1]][[refnum]] <- tmp.sim.out[[1]]

sim.list[[2]][[refnum]] <- tmp.sim.out[[2]]

tmp.sim.out=NULL

cat("", "\n")

cat("ended processing reference :", refnum, refvec2[refnum],paste(Sys.time(),", total time :"),Sys.time()-StartTime, "\n")

}

}

TotalRunTime=Sys.time()-StartTime

TotalRunTime

for(refnum in 1:length(refvec2)) {

sim.list[[1]][[refnum]] $reference<- refvec2[refnum]

}

sim.out=do.call("rbind", sim.list[[1]])

Refs=refvec2

for(i in 1:length(Refs)){

Refs[i]

tmpRef=read.csv2(paste("H:/Data/am_eu_bugs/species_",Refs[i],".csv", sep=""), header=T)

tmpNSp=nrow(tmpRef)

tmpNYr=length(sim.out[sim.out$param=="n[]" & sim.out$reference==as.character(tmpRef$refvec[1]),"param"])/tmpNSp

sim.out[sim.out$param=="n[]" & sim.out$reference==as.character(tmpRef$refvec[1]),"sp"] <- rep(as.character(tmpRef$spvec),tmpNYr)

sim.out[sim.out$param=="n[]" & sim.out$reference==as.character(tmpRef$refvec[1]),"yr"] <- rep(1:tmpNYr,each=length(tmpRef$spvec))

}

write.csv2(sim.out,"sim.out_w_n.csv", row.names=F)

head(sim.out[sim.out$sp%in%c("Redhead.Pochard", "Les.scaup.Tufted.duck"),])

spvec=names(summary(melt1$ variable))

diving=spvec[spvec%in%c("Redhead.Pochard", "Les.scaup.Tufted.duck")]

dabbling=spvec[spvec!="Redhead.Pochard"&spvec!="Les.scaup.Tufted.duck"]

sim.out[sim.out$sp%in%dabbling,"sp.group"] <- "Dabbling"

sim.out[sim.out$sp%in%diving,"sp.group"] <- "Diving"

sim.out.sp=sim.out[!is.na(sim.out$sp),]

for(i in 1:length(spvec)) sim.out.sp[sim.out.sp$sp==spvec[i],"spf"] <- i

sim.out.sp$spf <- factor(sim.out.sp$spf,levels=1:length(spvec), labels=spvec)

sim.out.sp

levels(sim.out.sp$param) ;levels(sim.out.sp$spf)

sim.out.sp[sim.out.sp$param%in%c("prop.intra[]","var.s[]"),c("param","Mean","sd","Continent","spf")]

var.para=paste("Sigma","[",1:length(spvec),",",1:length(spvec),"]", sep="") # params of variances

param.vec=names(sim.list[[2]][[i]]$ mean)

param.vec=param.vec[param.vec%in%c("prop.intra","intra","env.var")]

param.mcmc.list=vector("list", length(param.vec))

param.mcmc.list2=vector("list", length(param.vec))

for(param.num in 1:length(param.mcmc.list)){

mcmc.coef = vector("list", length(Refs))

for(ref.num in 1:length(Refs)) { ## gather mcmc chains of chosen parameter from each ref

tmp.mcmc=data.frame(sim.list[[2]][[ref.num]][[8]][[ param.vec[param.num] ]])

tmpsp=as.vector(sim.out.sp[sim.out.sp$reference==Refs[ref.num],"spf"])

names(tmp.mcmc) <- names(tapply(tmpsp,tmpsp,length))

tmp.mcmc$Continent <- sim.out.sp[sim.out.sp$reference==Refs[ref.num],"Continent"][1]

#tmp.mcmc=tmp.mcmc[sample(1:nrow(tmp.mcmc),10),] # NOTE! sub-sample for lighter dataset in order to adjust Fig !!!!!

tmp.mcmc2=melt(tmp.mcmc,id="Continent")

mcmc.coef[[ref.num]] <- tmp.mcmc2

mcmc.coef[[ref.num]]$ref <- Refs[ref.num]

}

mcmc.coef <- do.call("rbind", mcmc.coef)

mcmc.coef[mcmc.coef$variable%in%dabbling,"Guild"] <- "Dabbling"

mcmc.coef[mcmc.coef$variable%in%diving,"Guild"] <- "Diving"

names(mcmc.coef)[2] <- "sp"

param.mcmc.list[[param.num]] <- mcmc.coef

} # end for param.num

length(param.mcmc.list)

param.vec

for(param.num in 1:length(param.vec)){

tmp.sp.vec=as.character(param.mcmc.list[[param.num]]$sp)

param.mcmc.list[[param.num]]$sp2<-factor(tmp.sp.vec,levels=c("Mallard",

"Pintail",

"Shoveler",

"Am.wigeon.Wigeon",

"GW.teal.Teal",

"BW.teal.Garganey",

"Redhead.Pochard",

"Les.scaup.Tufted.duck"),

labels=c("Mallard",

"Pintail",

"Shoveler",

"Wig.|Am.w.",

"Teal|Gw.t.",

"Gar.|Bw.t.",

"Poc.|Redh.",

"T.d.|L.sc."))

param.mcmc.list[[param.num]]$Param <- param.vec[param.num]

} # end for param.num

######## Fig. 3, black and white

param.mcmc.list3=do.call("rbind",param.mcmc.list)

param.mcmc.list4=melt(param.mcmc.list3, id.vars=c("Continent","ref","Guild","sp2","Param"), measure.vars="value")

param.mcmc.list5=cast(param.mcmc.list4, Continent + Guild + ref + variable + sp2 + Param ~ ., mean)

names(param.mcmc.list5)[names(param.mcmc.list5)=="(all)"] <- "Value"

wid=7

#Cairo(width = wid, height = wid*21/29.7, file="Fig3.png", type="png", pointsize=12,

# bg = "transparent", canvas = "white", units = "in", dpi = 200)

x11(width = wid, height = wid*21/29.7)

ggplot() +

facet_grid(Param~sp2, scales = "free") +

geom_boxplot(data=param.mcmc.list5, mapping=aes(x=Continent,y=Value, fill=Continent),outlier.shape = NULL,outlier.colour = "darkgray", outlier.size = 1.5) +

#scale_y_continuous(limits = quantile(param.mcmc.list5$value, c(0.1, 0.9))) +

scale_fill_grey(start = 0.5, end = 0.8) +

ylab("Value") +

theme(axis.title.x = element_blank(), #element_text(face="bold", colour="black", size=10)

axis.text.x = element_blank(),axis.ticks.x = element_blank()) + # element_text(angle=45, vjust=0.5, size=10, colour="black")

theme(axis.title.y = element_text(face="bold", colour="black", size=10, vjust=.75),

axis.text.y = element_text(angle=0, vjust=NULL, hjust=NULL, size=10, colour="black")) +

#theme(legend.position="none")

theme(legend.position="bottom")

#dev.off()

cast(param.mcmc.list5, Continent + Param ~ ., range, subset=Param=="prop.intra")

######## Fig. 2, black and white

wid=4

x11(width = wid*21/29.7, height = wid)

ggplot() +

facet_grid(Param~Guild, scales = "free_y") +

geom_boxplot(data=param.mcmc.list5, mapping=aes(x=Continent,y=Value, fill=Continent),outlier.shape = NULL,outlier.colour = "darkgray", outlier.size = 1.5) +

#scale_y_continuous(limits = quantile(param.mcmc.list5$value, c(0.1, 0.9))) +

scale_fill_grey(start = 0.5, end = 0.8) +

ylab("Value") +

theme(axis.title.x = element_blank(), #element_text(face="bold", colour="black", size=10)

axis.text.x = element_blank(),axis.ticks.x = element_blank()) + # element_text(angle=45, vjust=0.5, size=10, colour="black")

theme(axis.title.y = element_text(face="bold", colour="black", size=10, vjust=.75),

axis.text.y = element_text(angle=0, vjust=NULL, hjust=NULL, size=10, colour="black")) +

#theme(legend.position="none")

theme(legend.position="bottom")

names(sim.out)

#[1] "param" "Mean" "sd" "X2.5." "X25." "X50." "X75." "X97.5." "Rhat" "n.eff" "paramInd" "sp" "Continent" "reference" "yr" "sp.group"

head(sim.out[sim.out$param%in%c("Dem.var[]"),c("param","Mean","sd","Continent","sp.group")])

Eu=sim.out[sim.out$param=="Sigma[]"&sim.out$paramInd!=var.para[1]&

sim.out$paramInd!=var.para[2]&

sim.out$paramInd!=var.para[3]&

sim.out$paramInd!=var.para[4]&

sim.out$paramInd!=var.para[5]&

sim.out$paramInd!=var.para[6]&

sim.out$paramInd!=var.para[7]&

sim.out$paramInd!=var.para[8]&

sim.out$Continent=="Europe",]

X11(width=4, height=4)

d <- density(Eu$Mean)

plot(d, xlim=c(-0.075,0.45), main="", xlab="Between-species covariance in communities", las=1, col="blue", lwd=2)

#polygon(d, col = "wheat")

Am=sim.out[sim.out$param=="Sigma[]"&sim.out$paramInd!=var.para[1]&

sim.out$paramInd!=var.para[2]&

sim.out$paramInd!=var.para[3]&

sim.out$paramInd!=var.para[4]&

sim.out$paramInd!=var.para[5]&

sim.out$paramInd!=var.para[6]&

sim.out$paramInd!=var.para[7]&

sim.out$paramInd!=var.para[8]&

sim.out$Continent=="North Am.",]

d <- density(Am$Mean)

lines(d, main="", xlab="", col="orange", lwd=2)

#polygon(d, col = "gray")

legend("topright", bty="n", c("Europe","North Am."), lty=1, lwd=2, col=c("blue","orange"))

X11(width=4, height=4)

Eu2=sim.out[sim.out$param%in%c("Dem.var[]")&sim.out$Continent=="Europe",c("param","Mean","Continent")]

X11(width=4, height=4)

d <- density(Eu2$Mean)

plot(d, xlim=c(0,1.1), main="", xlab="Demographic variance", las=1, col="blue", lwd=2)

#polygon(d, col = "wheat")

Am2=sim.out[sim.out$param%in%c("Dem.var[]")&sim.out$Continent=="North Am.",c("param","Mean","Continent")]

d <- density(Am2$Mean)

lines(d, main="", xlab="", col="orange", lwd=2)

#polygon(d, col = "gray")

legend("topright", bty="n", c("Europe","North Am."), lty=1, lwd=2, col=c("blue","orange"))

###### 2nd Bayes, meta-analysis on posteriors for estimating continent and guild effects

NITER=20000

NTHIN=20

DEBUG=T

param.vec

param.vec2=paste(param.vec,"[]", sep="")

### Continent effects, mixed model, "site" is random term

sink("Am_Eu_vl_model_2.r")

cat("

model{

beta ~ dnorm(0, 0.0001)

tau.site <- pow(sd.site,-2)

sd.site ~ dunif(0,10)

mu ~ dnorm(0, 0.0001)

tau.i <- pow(sd.i,-2)

sd.i ~ dunif(0,10)

for(i in 1:nsite){

alpha[i] ~ dnorm(mu,tau.site)

}

for(i in 1:nobs){

y[i] ~ dnorm(mu.y[i],tau.psd[i])

tau.psd[i] <- pow(psd[i],-2)

mu.y[i] <- alpha[sitevec[i]] + beta*continent[i] + eps.i[i]

eps.i[i] ~ dnorm(0,tau.i)

}

} # end model

",fill=TRUE)

sink()

dat.list <- vector("list", length(param.vec))

for(i in 1:length(param.vec)){

tmp1=sim.out.sp[sim.out.sp$param==param.vec2[i],c("param","Mean","sd","Continent","reference","sp.group","spf")]

tmp1$continent <- as.numeric(factor(tmp1$Continent))-1

tmp1$guild <- as.numeric(factor(tmp1$sp.group))-1

tmp1$sitevec <- as.numeric(factor(tmp1$reference))

dat.list[[i]] <- list(continent=tmp1$continent, guild=tmp1$guild, sitevec=tmp1$sitevec, y=tmp1$Mean, psd=tmp1$sd, nsite=length(tapply(tmp1$sitevec,tmp1$sitevec,length)), nobs=nrow(tmp1))

}

inits=list(

list(mu=rnorm(1,0,1), alpha=rnorm(dat.list[[1]]$nsite,0,1), beta=rnorm(1,0,1), sd.site=runif(0,10), sd.i=runif(0,10), eps.i=rnorm(nrow(tmp1),0,1)),

list(mu=rnorm(1,0,1), alpha=rnorm(dat.list[[1]]$nsite,0,1), beta=rnorm(1,0,1), sd.site=runif(0,10), sd.i=runif(0,10), eps.i=rnorm(nrow(tmp1),0,1)),

list(mu=rnorm(1,0,1), alpha=rnorm(dat.list[[1]]$nsite,0,1), beta=rnorm(1,0,1), sd.site=runif(0,10), sd.i=runif(0,10), eps.i=rnorm(nrow(tmp1),0,1)),

list(mu=rnorm(1,0,1), alpha=rnorm(dat.list[[1]]$nsite,0,1), beta=rnorm(1,0,1), sd.site=runif(0,10), sd.i=runif(0,10), eps.i=rnorm(nrow(tmp1),0,1)) #4th intits

)

save.para=c("beta")

sim.list2=vector("list", length(param.vec))

mcmc_objects1=vector("list", length(param.vec))

#sim.list2[[1]]<-bugs(data=dat.list[[1]], inits=inits, parameters.to.save=save.para, n.iter=NITER, n.thin=NTHIN, n.burnin=floor(NITER / 2), #

# model.file="H:/Data/am_eu_bugs/Am_Eu_vl_model_2.r", n.chains=length(inits), debug=DEBUG)

for(i in 1:length(param.vec)){

tmp.sim <- jags(data=dat.list[[i]], inits=inits, parameters.to.save=save.para, n.iter=NITER, n.thin=NTHIN, n.burnin=floor(NITER / 2), #

model.file="H:/Data/am_eu_bugs/Am_Eu_vl_model_2.r", n.chains=length(inits))

sim.list2[[i]] <- data.frame(Comparison="Continent",param.vec[i],tmp.sim$ BUGSoutput$ summary)

mcmc_objects1[[i]] <- tmp.sim

tmpvec=as.vector(mcmc_objects1[[i]]$ BUGSoutput$ sims.list$ beta)

if(mcmc_objects1[[i]]$ BUGSoutput$mean$beta>0){

Pr=length(tmpvec[tmpvec>0])/length(tmpvec)

} else{

Pr=length(tmpvec[tmpvec<0])/length(tmpvec)

}

sim.list2[[i]]$Prob<-c(Pr,NA)

} # end i

sims.para2 <- do.call("rbind",sim.list2)

#traceplot(mcmc_objects1[[1]], mfrow=c(4,4))

### Guild effect, Europe, mixed model, "site" is random term

sink("Am_Eu_vl_model_3.r")

cat("

model{

beta ~ dnorm(0, 0.0001)

tau.site <- pow(sd.site,-2)

sd.site ~ dunif(0,10)

mu ~ dnorm(0, 0.0001)

tau.i <- pow(sd.i,-2)

sd.i ~ dunif(0,10)

for(i in 1:nsite){

alpha[i] ~ dnorm(mu,tau.site)

}

for(i in 1:nobs){

y[i] ~ dnorm(mu.y[i],tau.psd[i])

tau.psd[i] <- pow(psd[i],-2)

mu.y[i] <- alpha[sitevec[i]] + beta*guild[i] + eps.i[i]

eps.i[i] ~ dnorm(0,tau.i)

}

} # end model

",fill=TRUE)

sink()

dat.list <- vector("list", length(param.vec))

for(i in 1:length(param.vec)){

tmp1=sim.out.sp[sim.out.sp$param==param.vec2[i]&sim.out.sp$Continent=="Europe",c("param","Mean","sd","Continent","reference","sp.group","spf")]

tmp1$guild <- as.numeric(factor(tmp1$sp.group))-1

tmp1$sitevec <- as.numeric(factor(tmp1$reference))

dat.list[[i]] <- list(continent=tmp1$continent, guild=tmp1$guild, sitevec=tmp1$sitevec, y=tmp1$Mean, psd=tmp1$sd, nsite=length(tapply(tmp1$sitevec,tmp1$sitevec,length)), nobs=nrow(tmp1))

}

inits=list(

list(mu=rnorm(1,0,1), alpha=rnorm(dat.list[[1]]$nsite,0,1), beta=rnorm(1,0,1), sd.site=runif(0,10), sd.i=runif(0,10), eps.i=rnorm(nrow(tmp1),0,1)),

list(mu=rnorm(1,0,1), alpha=rnorm(dat.list[[1]]$nsite,0,1), beta=rnorm(1,0,1), sd.site=runif(0,10), sd.i=runif(0,10), eps.i=rnorm(nrow(tmp1),0,1)),

list(mu=rnorm(1,0,1), alpha=rnorm(dat.list[[1]]$nsite,0,1), beta=rnorm(1,0,1), sd.site=runif(0,10), sd.i=runif(0,10), eps.i=rnorm(nrow(tmp1),0,1)),

list(mu=rnorm(1,0,1), alpha=rnorm(dat.list[[1]]$nsite,0,1), beta=rnorm(1,0,1), sd.site=runif(0,10), sd.i=runif(0,10), eps.i=rnorm(nrow(tmp1),0,1)) #4th intits

)

save.para=c("beta")

sim.list3=vector("list", length(param.vec))

mcmc_objects2=vector("list", length(param.vec))

for(i in 1:length(param.vec)){

tmp.sim <- jags(data=dat.list[[i]], inits=inits, parameters.to.save=save.para, n.iter=NITER, n.thin=NTHIN, n.burnin=floor(NITER / 2), #

model.file="H:/Data/am_eu_bugs/Am_Eu_vl_model_3.r", n.chains=length(inits))

sim.list3[[i]] <- data.frame(Comparison="Guild",Continent="Europe",param.vec[i],tmp.sim$ BUGSoutput$ summary)

mcmc_objects2[[i]] <- tmp.sim

tmpvec=as.vector(mcmc_objects2[[i]]$ BUGSoutput$ sims.list$ beta)

if(mcmc_objects2[[i]]$ BUGSoutput$mean$beta>0){

Pr=length(tmpvec[tmpvec>0])/length(tmpvec)

} else{

Pr=length(tmpvec[tmpvec<0])/length(tmpvec)

}

sim.list3[[i]]$Prob<-c(Pr,NA)

} # end i

sims.para3 <- do.call("rbind",sim.list3)

### Guild effect, North Am., linear model; "site" is factor, only two sites

sink("Am_Eu_vl_model_4.r")

cat("

model{

alpha0 ~ dnorm(0, 0.0001)

beta ~ dnorm(0, 0.0001)

alpha ~ dnorm(0,0.0001)

tau.i <- pow(sd.i,-2)

sd.i ~ dunif(0,10)

for(i in 1:nobs){

y[i] ~ dnorm(mu.y[i],tau.psd[i])

tau.psd[i] <- pow(psd[i],-2)

mu.y[i] <- alpha0 + alpha*site[i] + beta*guild[i] + eps.i[i]

eps.i[i] ~ dnorm(0,tau.i)

}

} # end model

",fill=TRUE)

sink()

dat.list <- vector("list", length(param.vec))

for(i in 1:length(param.vec)){

tmp1=sim.out.sp[sim.out.sp$param==param.vec2[i]&sim.out.sp$Continent=="North Am.",c("param","Mean","sd","Continent","reference","sp.group","spf")]

tmp1$guild <- as.numeric(factor(tmp1$sp.group))-1

tmp1$site <- as.numeric(factor(tmp1$reference))-1

dat.list[[i]] <- list(continent=tmp1$continent, guild=tmp1$guild, site=tmp1$site, y=tmp1$Mean, psd=tmp1$sd, nobs=nrow(tmp1))

}

inits=list(

list(alpha0=rnorm(1,0,1), alpha=rnorm(1,0,1), beta=rnorm(1,0,1), sd.i=runif(0,10), eps.i=rnorm(nrow(tmp1),0,1)),

list(alpha0=rnorm(1,0,1), alpha=rnorm(1,0,1), beta=rnorm(1,0,1), sd.i=runif(0,10), eps.i=rnorm(nrow(tmp1),0,1)),

list(alpha0=rnorm(1,0,1), alpha=rnorm(1,0,1), beta=rnorm(1,0,1), sd.i=runif(0,10), eps.i=rnorm(nrow(tmp1),0,1)),

list(alpha0=rnorm(1,0,1), alpha=rnorm(1,0,1), beta=rnorm(1,0,1), sd.i=runif(0,10), eps.i=rnorm(nrow(tmp1),0,1)) #4th intits

)

save.para=c("beta")

sim.list4=vector("list", length(param.vec))

mcmc_objects3=vector("list", length(param.vec))

for(i in 1:length(param.vec)){

tmp.sim <- jags(data=dat.list[[i]], inits=inits, parameters.to.save=save.para, n.iter=NITER, n.thin=NTHIN, n.burnin=floor(NITER / 2), #

model.file="H:/Data/am_eu_bugs/Am_Eu_vl_model_4.r", n.chains=length(inits))

sim.list4[[i]] <- data.frame(Comparison="Guild",Continent="North Am.",param.vec[i],tmp.sim$ BUGSoutput$ summary)

mcmc_objects3[[i]] <- tmp.sim

tmpvec=as.vector(mcmc_objects3[[i]]$ BUGSoutput$ sims.list$ beta)

if(mcmc_objects3[[i]]$ BUGSoutput$mean$beta>0){

Pr=length(tmpvec[tmpvec>0])/length(tmpvec)

} else{

Pr=length(tmpvec[tmpvec<0])/length(tmpvec)

}

sim.list4[[i]]$Prob<-c(Pr,NA)

} # end i

sims.para4 <- do.call("rbind",sim.list4)

write.csv2(sims.para2,"Bayes2_continent.csv")

write.csv2(rbind(sims.para3, sims.para4),"Bayes2_guild.csv")

save.image("H:/Data/am_eu_bugs/am_eu_BUGS_20t_1.7.2016.RData")
